# Rapid radiation of humans in South America after the last glacial maximum: A radiocarbon-based study

**DOI:** 10.1371/journal.pone.0236023

**Published:** 2020-07-22

**Authors:** Luciano Prates, Gustavo G. Politis, S. Ivan Perez

**Affiliations:** 1 Consejo Nacional de Investigaciones Científicas y Técnicas, Buenos Aires, Argentina; 2 División Arqueología, Facultad de Ciencias Naturales y Museo, Universidad Nacional de La Plata, La Plata, Argentina; 3 Instituto de Investigaciones Arqueológicas y Paleontológicas del Cuaternario Pampeano (CONICET), Universidad Nacional del Centro de la Provincia de Buenos Aires, Olavarría, Argentina; 4 División Antropología, Facultad de Ciencias Naturales y Museo, Universidad Nacional de La Plata, La Plata, Argentina; Max Planck Institute for the Science of Human History, GERMANY

## Abstract

The early peopling of the Americas has been one of the most hotly contested topics in American anthropology and a research issue that draws archaeologists into a multidisciplinary debate. In South America, although the background data on this issue has increased exponentially in recent decades, the core questions related to the temporal and spatial patterns of the colonization process remain open. In this paper we tackle these questions in the light of the quantitative analysis of a screened radiocarbon database of more than 1600 early dates. We explore the frequency of radiocarbon dates as proxies for assessing population growth; and define a reliable and statistically well supported lower chronological bound (not to the exact date) for the earliest human arrival. Our results suggest that the earliest chronological threshold for the peopling of South America should be between 16,600 and 15,100, with a mean estimated date ~ 15,500 cal BP (post Last Glacial Maximum). Population would have grown until the end of Antarctic Cold Reversal stadial ~12,500 cal BP at the time of the main extinctions of megafauna–, when the increase rate slows, probably as a result of the changes that occurred in the trophic niche of humans.

## Introduction

The early peopling of the Americas has been one of the most hotly contested topics in American anthropology [1–6] and a research issue that draws archaeologists into a multidisciplinary debate [7]. In South America, although the quantity and quality of data related to this issue has increased exponentially in recent decades and fostered a growing interest in comprehensive and multi-proxy data [8], most of the core questions related to the timing of human arrival and temporal patterns of the colonization process still remain open. The time of the early peopling has been probably the main controversial point in the debate. From the early 1960s onward there have been numerous hypotheses that can be summarised in accordance with the three main competing models for the early peopling of the Americas [1, 5, 9]. First, the Short Chronology model which proposes that humans arrived to North America not long before 13,000 calibrated years before present (from now on referred to as cal BP), and not before ~12.900 cal BP to South America; its defenders consider the “Clovis Complex” as first widespread archaeological culture in North America [10]. The second model (“Clovis Second”) asserts an intermediate antiquity and proposes a pre-Clovis but post-Last Glacial Maximum (LGM) peopling of the continent (~18,500 to 13,000 BP); implying a human entry into South America a short time later [9]. Finally, the Long Chronology model which proposes a para- or pre-LGM entry and argues for a pre-18,000 cal BP peopling of South America [11, 12]. The actual time of the first arrival of humans has not been directly addressed, but approximately derived from the age of the alleged oldest unambiguous archaeological evidence. So, scholars who validate pre-18,000 cal BP sites are natural defenders of the Long Chronology model, and the opponents are usually in favor either of the Short or of the Intermediate Chronology models [5, 9, 10, 13–15].

Though these competing models initially emerged from archaeology, anthropological studies based on genetic data–especially analysis of substitution rate and variability in autosomal, mtDNA, and Y chromosome–have made significant contributions in this field over the last decade [16–22]. Based on molecular substitution rates, recent research suggests–independently of radiocarbon dates- humans to have entered America from Beringia between 19,500 and 14,000 cal BP [16, 19, 21], and South America between 18,500 and 15,000 cal BP [17, 22]. Additionally, taking into account the differences in genetic variability in current and ancient human samples, the previous studies indicate that the initial migration occurred in a quite rapid dispersion mainly following the Pacific coast. Despite the large errors of genetic approaches due to methodological limitations, they seem to give support to Intermediate Chronology model (see Llamas et al. [16] for a discussion of the TMRCA -times to most recent common ancestor- bounds for each of the Native American haplogroups based on different molecular calibrations).

Here, we discuss the process of the early peopling of South America in the light of a quantitative-based analysis of early archaeological dates, which has not been implemented in the area so far. Based on the idea that the earliest archaeological site, no matter how early, is unlikely to be the earliest because of the small and biased archaeological sample [23, 24], we seek to estimate a reliable and statistically well supported chronological bound (not to the exact date) for the earliest human arrival. With that purpose we have employed a formal statistical approach to estimate confidence intervals for the earliest peopling and to explore the temporal pattern of the older end of the distribution of calibrated dates. We also explore the frequency of radiocarbon dates as proxies for assessing population growth at the early stages of the colonization process and compare our results with the expectations of the main confronting models explaining the early peopling of all-Americas: Short, Intermediate and Long chronology.

## Materials and methods

### Preliminary validation of the dataset

**Fig 1:**
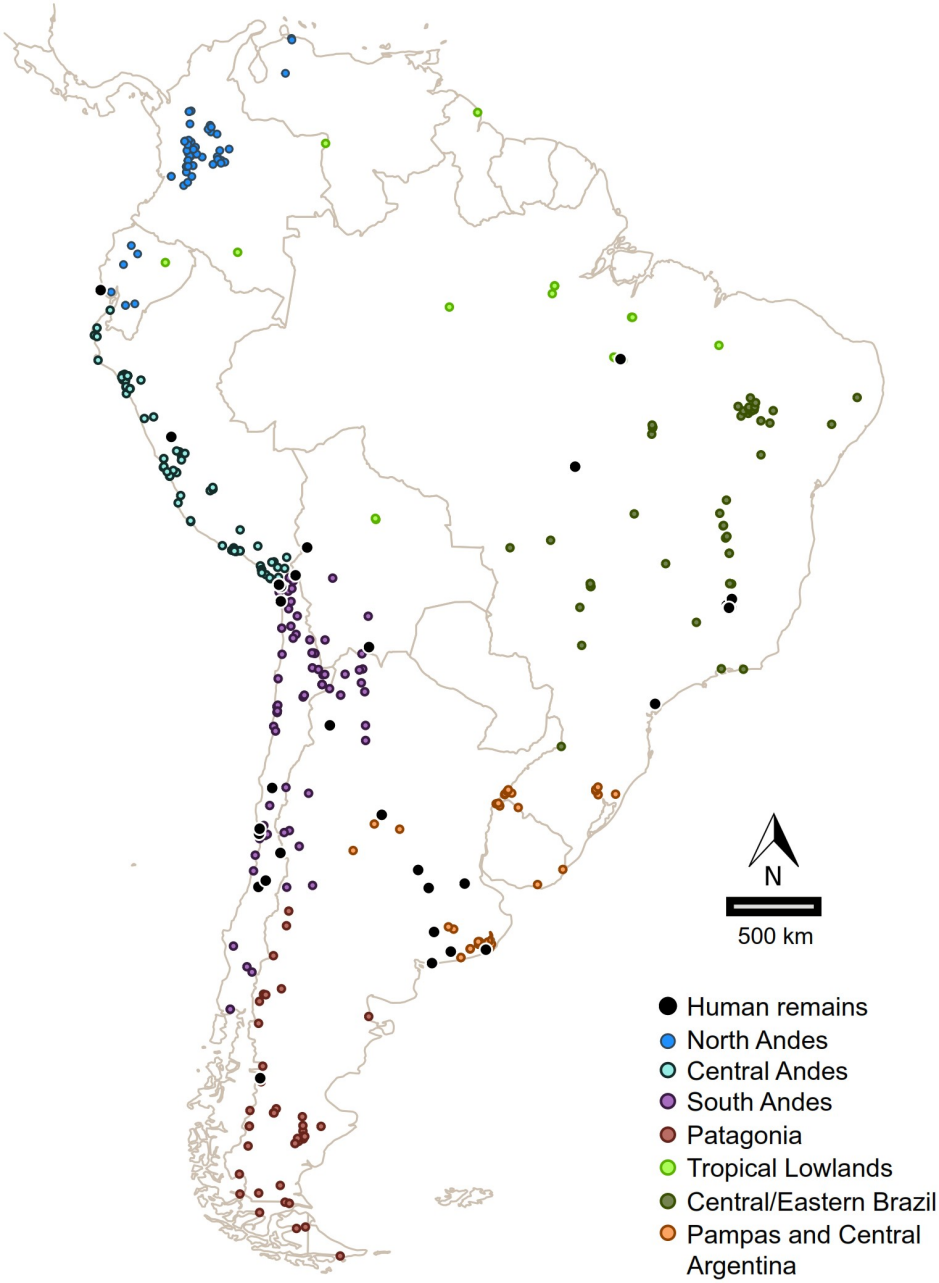
Distribution of archaeological sites with validated dates in South America. Map generated with the package sp and rworldmap for R software, and regions defined based on geographical and archaeological criteria [46, 47].

Dates and sites included in the analysis (Fig 1) meet the commonly stated requirements for securely diagnosing and dating past human activity at an archaeological location. The dated material must have clear stratigraphic and contextual association with unambiguous traces of humans (artefacts or skeletons) in well-defined geological deposits, and must have reliably measured radiometric ages [25]. To exclude dates from the sample to be analyzed, a supplementary set of criteria was considered. We exclude from our analysis: a) dates with statistically imprecise radiometric measurement (i.e. with errors larger than 350 years); b) dates not based on single objects of human or cultural carbon (human remains, wood charcoal, fruit/seed remains, or bones/teeth of prey animals); c) samples with weak evidence for an association with human agency and/or with poor/ambiguous description of the archaeological context; d) dates obtained from samples presumed to be contaminated, e) dates rejected or called into question by the authors of the primary publication, f) dates with ambiguous results from re-dating, and g) singular, unreplicated dates, in cases these are the oldest in their the region. Beside using the whole dataset, and with the aim of evaluating the effect of other potential biases (among others, reservoir effect and old-wood effects), we additionally run the analyses excluding dates on shell, dates on charcoal, dates on wood, dates with errors larger than 200 years. The approach of this paper should not be seen as a means to define the exact date of the peopling of South America, but as a means to define a reliable and statistically well supported chronological bound for that first arrival.

### Estimation of the initial time of the peopling

The colonization process of a new territory implies a variable time of low population density, and thus a variable time of exceptionally low levels of preservation/visibility in the archaeological record [23, 26]. So it can be reasonably assumed that the oldest documented archaeological record occurs sometime after the actual first arrival. The question is how long human occupation characteristically takes to become archaeologically detectable in a typical field sampling regime. To answer this question for South America, we estimated confidence intervals for this arrival time using the information in the older tail of the distribution of calibrated dates, and modeled the uncertainties imposed by incomplete sampling and by the formation processes of the archaeological record. We applied six non-Bayesian methods developed to estimate origination and extinction dates of fossil taxa in the geological record [27–32], which is subject to the same principles we require to evaluate the first arrival of humans to a new territory. We focused here on one of the methods recommended by Saltré and collaborators [33], Marshall’s method [34], not only because it’s shown one of the highest model performance and accuracy for inferring extinction time of species (see comparison in 31) but also because it allows for estimating the largest confidence interval on the lower (older) bound, and thus gives the maximum estimate for the earliest arrival time, which is relevant to our distinction between pre-LGM and post-LGM scenarios of peopling. Marshall’s method provides a generalization of Strauss and Sadler’s [27] approach, where the 95% confidence interval around the estimated time of initial peopling depends on the time range and number of records [32]. In these methods, the time for the earlier peopling is a fraction of the observed range of dates, which depends on the number of dates, and its estimation requires an observed distribution of dates and a site/date recovery potential function. In this method, the 95% confidence interval may be viewed as the more representative gap calculated from the weighted average of the gaps between finds, which take into account the number of gaps and the potential preservation [32]. The estimates were calculated in R [34] using the algorithm proposed and provided by Saltré and collaborators [33].

### Exploring the early demographic change using summed probabilities

To explore the early population growth and dispersion of humans in South America we first used the Monte-Carlo Sum Probability Distribution method (MCSCPD method) proposed by Shennan et al. [35] and Timpson et al. [36]. This method was used to compare the Summed Calibrated Probability Density (SCPD) of radiocarbon dates, which have been suggested to be proxies for population size [37] or the population density [14], with a simulated null model expectation [36, 38]. The SCPD was reconstructed using calibrated radiocarbon data binned by site-phase in intervals of 200 years, ensuring at least a 200-year gap between each phase at each site. The use of these bins allows averaging the calibrated dates from the same site-phase, so as to ensure that each site-phase has the same weight in the final SCPD. All site-phase distributions are then summed and normalised to unity, yielding the empirical SCPD. Following this, two null models of exponentially and logistically growing population were fitted to the empirical SCPD. The exponential model was fit using the option 'exponential' implemented in the function modelTest from the package rcarbon package for R [39], whereas the logistic model was fit using the option 'custom' implemented in the same package. The parameters of the logistic model were estimated using the “nls” function of the package stats for R [33], employing a nonlinear (weighted) least-square to fit the logistic curve to the empirical SCPD. These null models were then used as the probability distribution for the Monte Carlo simulator to generate larger sets of simulated radiocarbon dates (e.g., 1,000 or 10,000 simulation in our work) and a 95% confidence interval around them. Significant deviations from the model expectation appear outside of this confidence interval. The MCSPD analysis was performed in the rcarbon package for R [39]. The relative fit of the SCPD (dependent variable) to each model (independent variable) was explored using a regression model and the R^2^ and an information-theoretic model selection criterion, Akaike (AIC [40]), using the lm and AIC functions of the stats package for R [34]. For each regression model, the AIC was calculated as -2*log-likelihood + k*npar, where *npar* is the number of parameters and *k* = 2.

The spatial heterogeneity in the population density and growth was explored using several approaches. First, density maps were produced with QGIS 3.4. [41], using a Kernel method to display dates density, estimated as the number of dates in a given location or site for a given interval, with larger numbers of clustered points resulting in higher densities. These intervals are of 2,000 years and the density maps were generated based on a bandwidth of 550 km and the quartic kernel shape. Additionally, SCPD curves of each geographical region were compared against each other to evaluate regional differences in the early population curve [36], using the permutation test proposed by Crema et al. [42] and implanted in the rcarbon package for R software [39]. Considering that uneven research efforts and ecological constraints could have generated unequal sampling across regions, geographical grouping was defined on both ecological and archaeological criteria. Finally, we used the spatial extension of the permutation test [38] to detect both positive and negative geographical deviations from the South American global rates of change in calibrated radiocarbon date and to observe the variation in growth rate in a continuous way in the geographical space. For this analysis we consider four intervals of 2,000 years, which represent a conservative time that insures an acceptable (spatial and temporal) representation of dates over South America.

## Results

### Radiocarbon dataset composition

With the aim of discussing both the time of the first human arrival, and the population growth process during the following millennia, the database analyzed here includes evidence older than 7000 ^14^C yrs BP. Considering that calibrate ages obtained using Calib 7.0.1 [43] and the function of the rcarbon package for R [39] were almost identical, we use the latter software and the SHCal13 calibration curve [44]. The main dataset was built in compliance with standard validation criteria and is composed of 1661 dates, (1543 made on cultural materials or related remains, and 118 on human bones/tooth) from 454 archaeological sites (see S1 Dataset and Fig 1). Although during recent decades several pre-15,000 cal BP archaeological sites/levels have been considered as presumptive candidates for the earliest evidence of a human presence in South America, they have not been included in our analysis (see excluded dates in S2 Dataset) because we consider that they do not meet standard validation requirements. Among the most renowned excluded sites are: Boqueirao da Pedra Furada, Vale da Pedra Furada, Toca do sitio do Meio, Toca da Tira Peia (Serra do Capivara sites, in Brazil), Santa Elina (Matogrosso, Brasil), Arroyo del Vizcaíno (Uruguay), Monte Verde I / Chinchihuapi II and the pre-14,500 cal BP levels of Chinchihuapi I, and Pilauco (Chile). The major weaknesses of the Piauí sites are the disputed origin of several of the alleged pieces of human evidence (stone tools and hearths) and the scarcity of taphonomic studies. In Monte Verde I / Chinchihuapi I and II the density of archaeological evidence older than 14,500 cal BP is extremely low and not conclusive enough to be considered as a clear signal of early humans. Even considering that all the recovered materials belong to ephemeral occupations, the evidence presented up to now is still weak and would need to be confirmed (as Dillehay et al. [45] have stated) and we preferred to keep the dates out for now. In the Pilauco site there are inconsistences in the geological provenience of the dated samples in the different publications which make it difficult to adjust the chronology of the associated materials. Again, it is not our intention to dismiss these sites in the context of a broader discussion about the peopling of South America, but specifically, for the purpose of this analysis and taking into account the above criteria, we excluded their radiocarbon dates from the main data base (for a discussion on the excluding of specific sites, see S1 Text).

However, since there is no universal acceptance of all sites/evidence and the filtering of dates entails the risk of both excluding evidence that may represent correlates of early occupations and including misassigned archaeological associations, we complementarily utilized databases other than our filtered one to evaluate how the results of some analyses might change; one of them more conservative–which does not include pre-13,000 cal BP dates–and one less conservative–which includes most of the pre-14,500 cal BP dates–(see S1 Fig).

### Oldest archaeological signal and estimation of the initial time of the peopling

**Fig 2:**
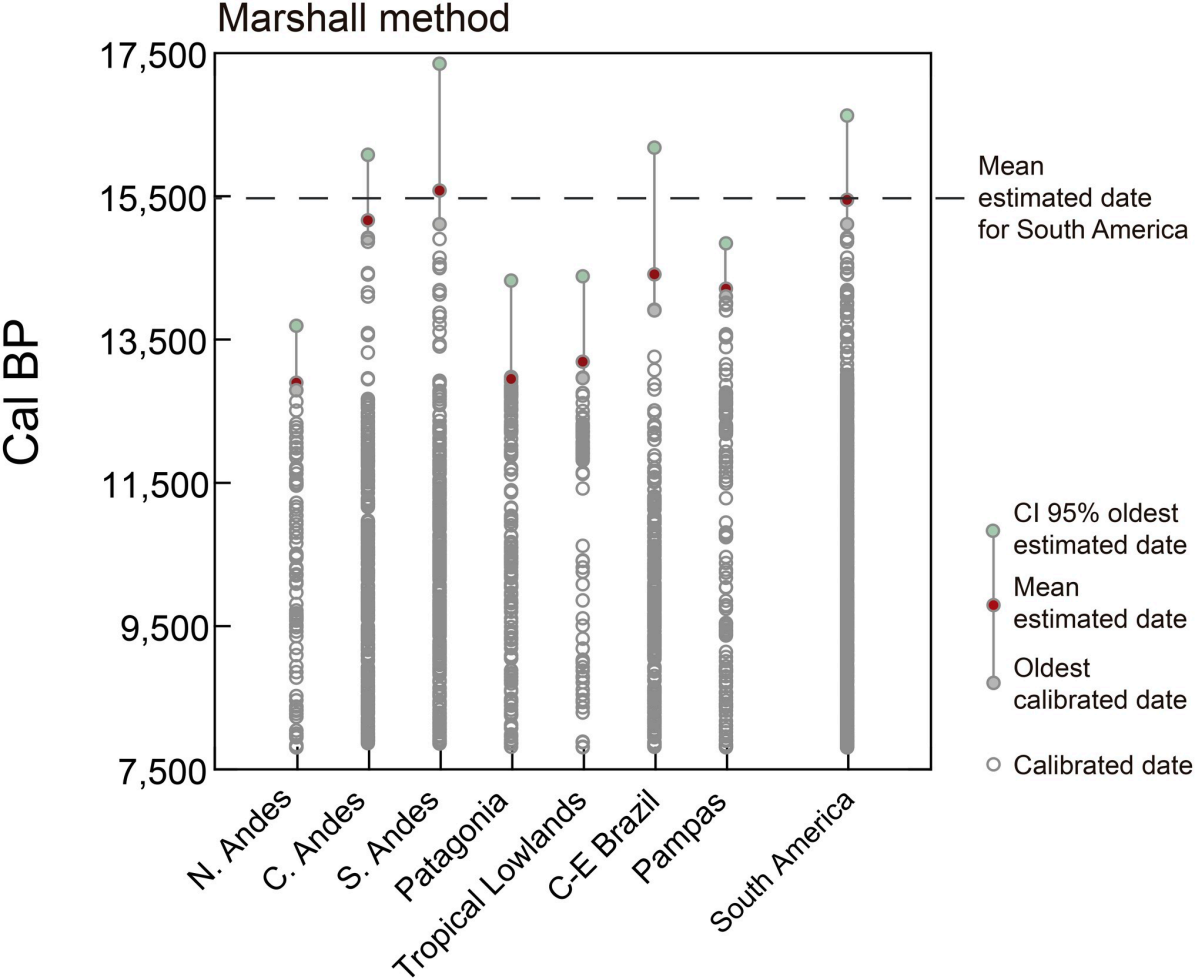
Estimation of first arrival for all the regions of South America using Marshall’s method. The oldest calibrated date for each region is shown in grey circles (Tequendama 1 -North Andes-, Huaca Prieta -Central Andes-, Monte Verde II -South Andes-, Cerro Tres Tetas 1 -Patagonia-, Caverna da Pedra Pintada -Tropical Lowlands-, Lapa do Boquete -Central-East Brazil-, Arroyo Seco 2 -Pampas-). The mean oldest estimated date is displayed in red circles, and grey lines represent a 95% confidence interval.

Based on our validation criteria, the oldest reliable archaeological evidence from South America came from Central Andes, South Andes, and Pampas (Huaca Prieta, Monte Verde, and Arroyo Seco 2) and were dated between15,100 and 14,000 cal BP. Nevertheless, since it is expected that the oldest recovered archaeological evidence is more recent than the actual time of first human arrival, a quantitative estimation of the time for the first arrival was performed by extrapolating back from the older end of the distribution of calibrated dates. The result of Marshall’s method [32] is displayed in Fig 2, where early peopling estimations are shown considering South American regions separately; grey lines represent a 95% confidence band around the estimated time of initial peopling. Initial arrival is estimated to have been in the range of: ~16,100–14,900 cal BP in Central Andes, ~17,300–15,100 cal BP in South Andes; ~16,200–13,900 cal BP in CE Brazil; ~14,400–13,000 cal BP in Tropical Lowlands; ~14,800–14,100 cal BP in Pampas; ~13,700–12,800 cal BP in North Andes, and ~14,300–13,000 cal BP in Patagonia (S1 Table). Full intervals are represented by grey lines in Fig 2 and they span the time between the oldest archaeological observations (grey circles), and the mean estimated initial arrival (red circles). If we consider South America as a whole, the statistically well supported lower chronological bound of first arrival was estimated between 16,600 and 15,100 cal BP. The last value can be considered the most conservative and recent estimation of the peopling of the region, whereas the first one is the maximum limit of this estimation. The mean estimated date for South America (dashed line in Fig 2) is ~ 15,500 cal BP and represents the most probable time for the first human arrival. Interestingly, this result varies little (~ 16,300–14,900) after removing the dates probably associated with other potential biases (dates on shell, dates on charcoal, dates on wood and singular-unreplicated oldest dates from a region) (see S2 Table) from the dataset.

### Summed probabilities, spatial distributions and the early population growth in South America

**Fig 3:**
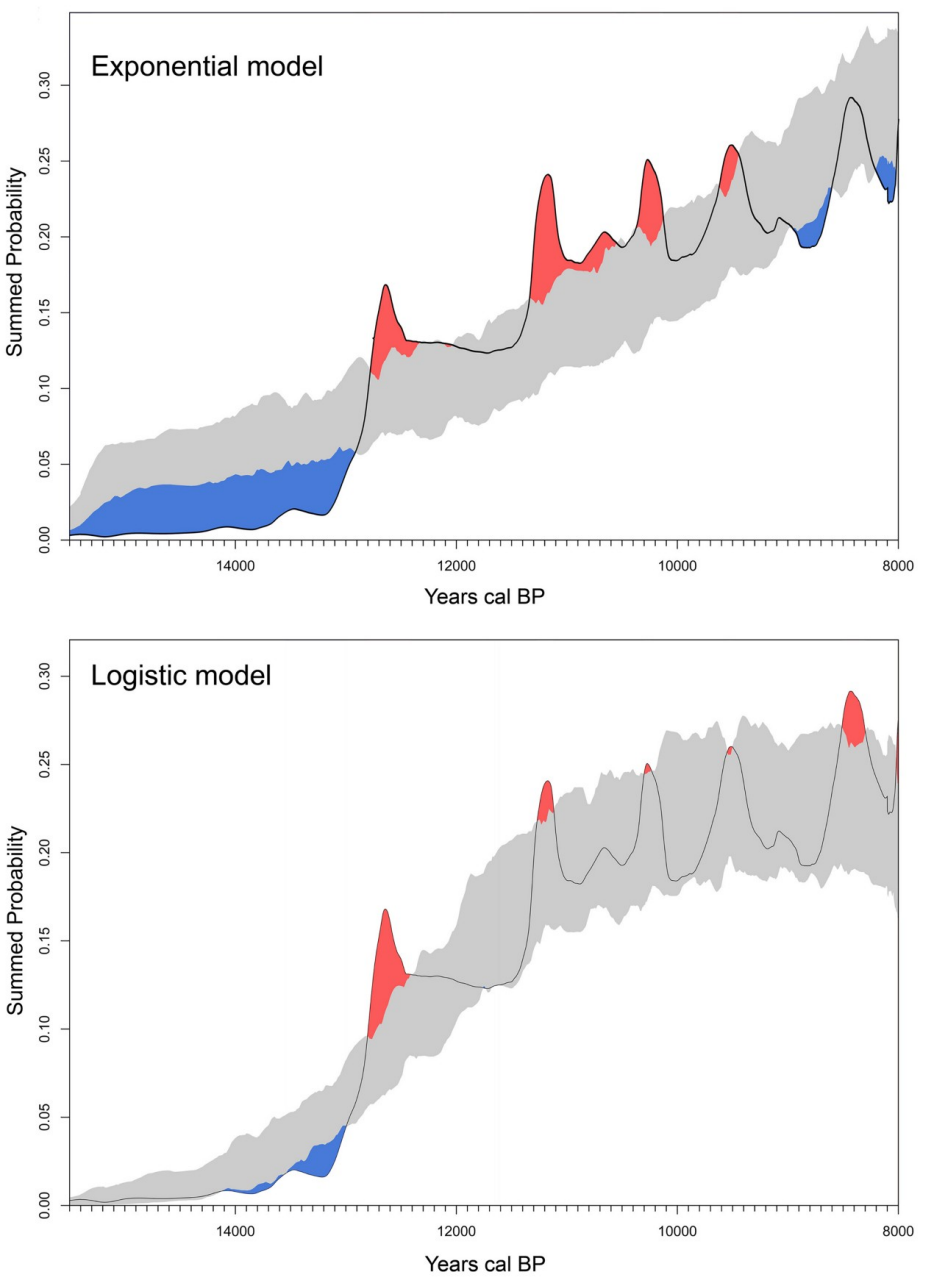
Summed probability distributions and Model Fit for all Radiocarbon dates. ^14^C Dates (black line), with fitted null model and its 95% confidence interval (grey-shaded area) for an exponential fit (top) and logistic fit (bottom). Red-shaded areas indicate regions that are above the expectation model’s confidence interval, whereas blue shading indicates regions below. Figure generated with rcarbon package of R [39].

Considering the SCPD displayed by the Fig 3, during the first period with human evidence in South America (15,100–13,500 cal BP) the intensity of the archaeological signal is extremely low. Then, the signal increases slowly from *ca*. 13,400, and rapidly between 13,200 and 12,700 cal BP. Between ca. 12,500 and 11,700 the rate decrease slows and ca. 11,300 increases rapidly again until 11,200 cal BP. Between *ca*. 11,000 cal BP and the younger end of the database’s temporal range the population growth stabilizes and a gradual increase is observed in the long term.

Fig 3 also shows the comparison of the SCPD against both the exponential (top) and logistic (bottom) demographic growth models. The former implies that population growth does not decline along time; whereas the latter is characterized by a slow-down as the carrying capacity, established by the available resources, is approached. The information-theoretic model selection approach [40] shows that the logistic fit is preferred over the exponential fit (AIC weight = 1.0, see Table 1).

**Table 1:** Model selection results for the two regression fitting models using all radiocarbon dates and only human dates.

	*Model*	*R^2^*	*logLik*	*AIC*	Δ	*w*
***All***	**Exponential**	0.7765	12715.43	-25424.87	8101.36	0.00
***dates***	***Logistic***	**0.9241**	**16766.12**	**-33526.23**	**0.00**	**1.00**
***Human***	**Exponential**	0.8304	30017.73	-60029.47	4168.31	0.00
***dates***	**Logistic**	**0.9027**	**32101.89**	**-64197.78**	**0.00**	**1.00**

R^2^ is the mean square degree of fit for the SPDs, logLik is the log-likelihood of the regression models, AIC is the calculated Akaike’s Information Criteria, Δ is the AIC difference and w is Akaike’s weight, or model probability that is calculated as exp (-0.5 * ΔAIC of each model) divided by the sum of these values for all models.

**Fig 4:**
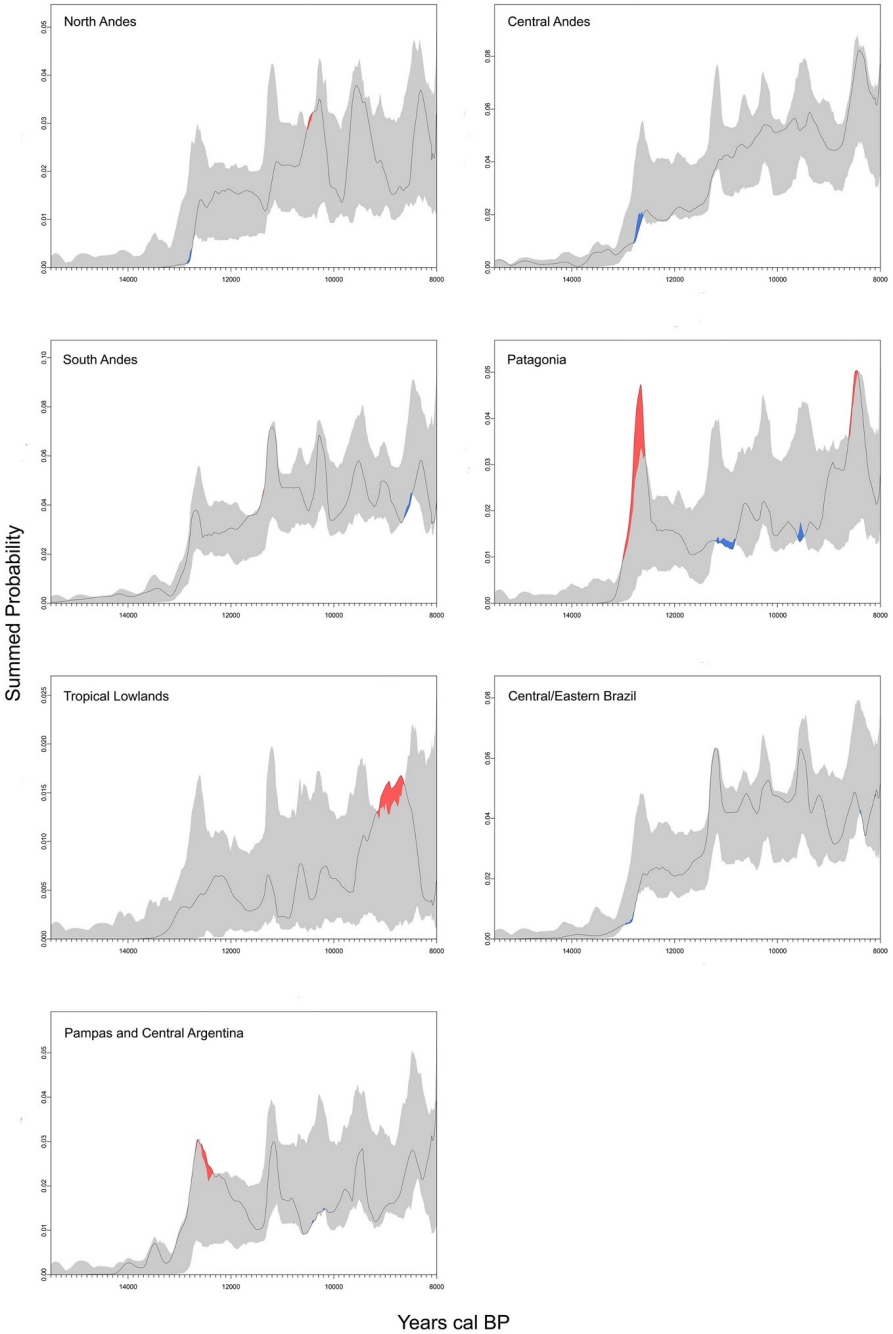
Summed probability distributions of radiocarbon dates from different regions. 14C Dates (black line), with permutation 95% confidence interval (grey-shaded area). Red-shaded areas indicate regions that are above the permutation confidence interval, whereas blue shading indicates regions below. Figure generated with rcarbon package for R [39].

The long-term growth pattern observed in the Fig 3 is, however, punctuated by spikes that take the SCPD curve outside the Monte Carlo-simulated logistic model’s 95% confidence interval, which implies they might not be due entirely to the effect of the calibration curve (not taken into account by the MCSCPD methodology). These spikes occur around 12,500, 11,200, 10,200, 9500 and 8400 cal BP (the first and the last ones seem to be the most significant) and, hence, they might be indicative of an irruptive growth dynamic, where the population grows so quickly that they overshoot their long-term carrying capacity. The permutation test comparing the regional curves allows observing that these peaks can also be related to over-sampling or over-dating particularly in Patagonia, for the most ancient sites (Fig 4), which could have generated or amplified the peak observed at ca. 12,500 cal BP in the SCPD curve (Fig 3). Fig 4 also shows that the main changes observed in the curve behave similarly in most of the regions of South America. As seen above regarding the estimated time for first arrival, no significant variations emerge from curves run with the whole dataset and with different filtering of dates (removing shell/charcoal/wood/singular, unreplicated dates, and with different values of error–less than 200 and less than 350 years, respectively). The most important variation is observed between 11,000 and 11,500 cal BP, where the peak displayed in the curve run with the whole dataset disappears after dates on shell charcoal and wood are removed (S2 Fig).

**Fig 5:**
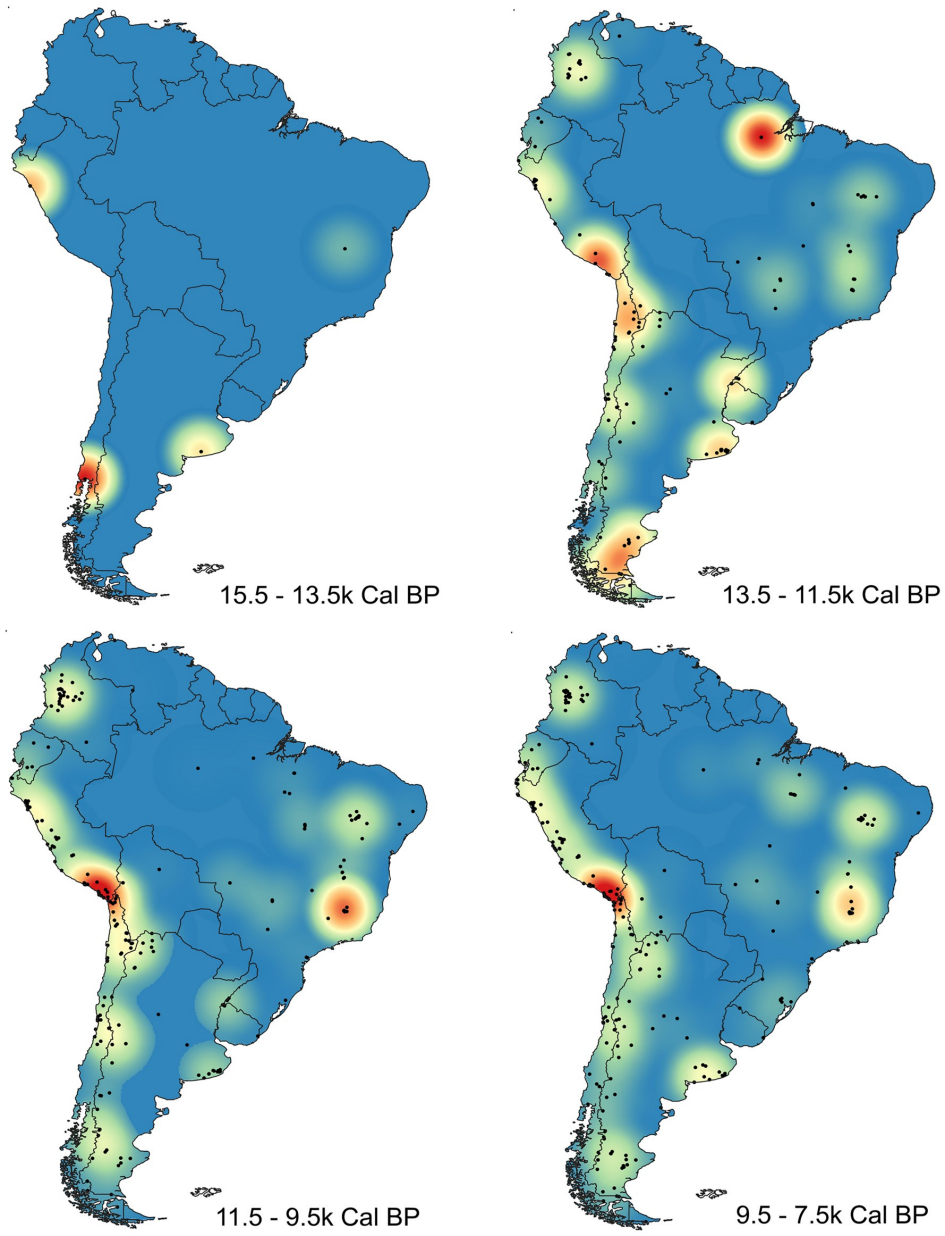
Maps of density of radiocarbon dates in SA in different periods. a) 15,500–13,500 cal BP; b) 13,500–11,500 cal BP; c) 11,500–9,500 cal BP; d) 9,500–7,500 cal BP. Map generated with QGIS version 3.4 (www.qgis.org).

From a geographic perspective, density maps of radiocarbon dates show that the archaeological signal during the first two millennia (ca. 15,100–13,500 cal BP) only appears in three main foci: central Andes, southern Andes, and Pampas (Fig 5). One thousand years later human evidence is still sparse but reaches different regions of South America (Southern and Central Andes, Pampas, Patagonia, and Central/Eastern Brazil) (Fig 5).

**Fig 6:**
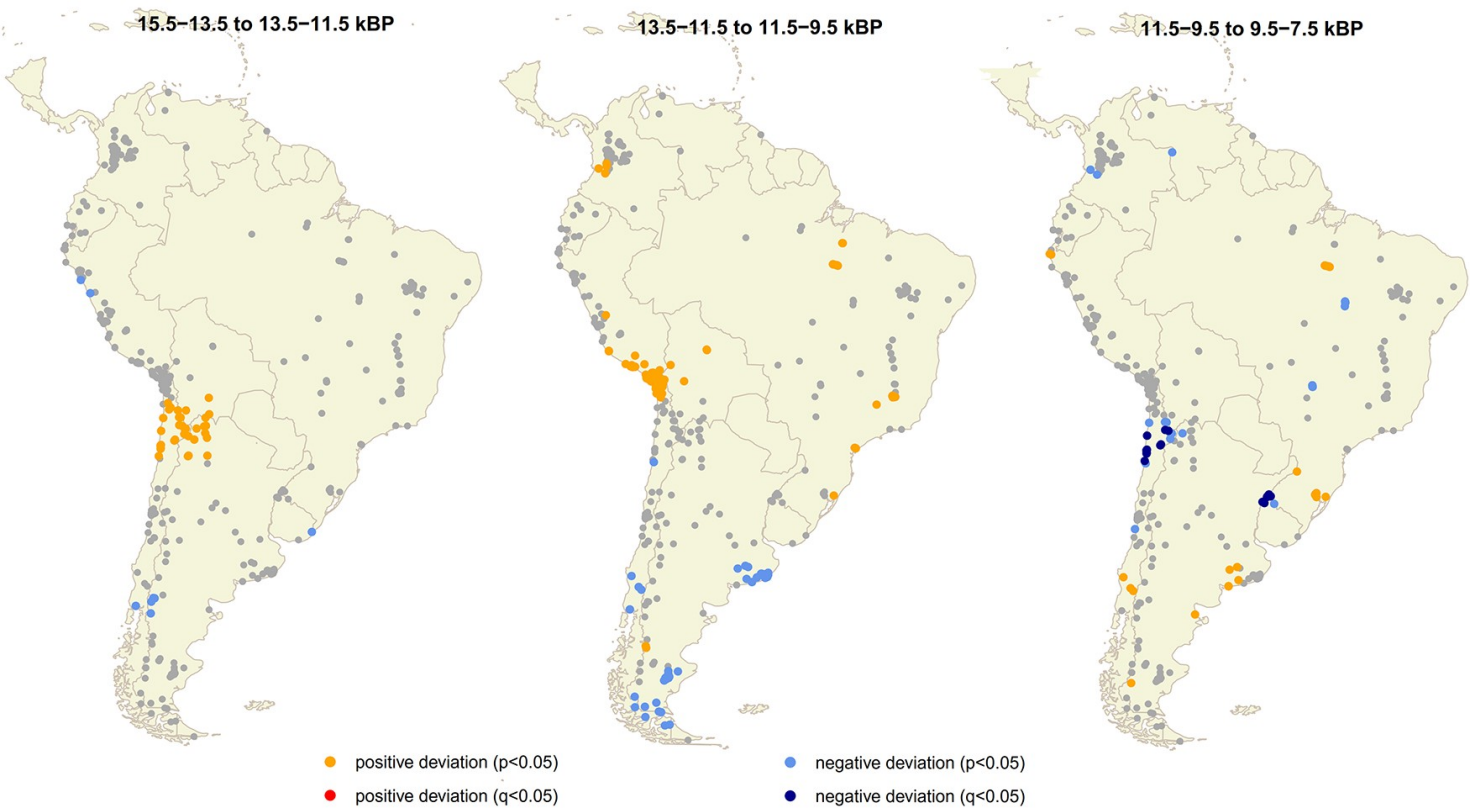
Spatial permutation test in South America for three transitions. The map displays the significance of the local geometric growth rate displayed in the S3 Fig. The three breaks and four intervals of 2,000 years were estimated on the SCPD curve with the rcarbon package for R [41].

Additionally, Fig 6 and S3 Fig display the relative changes in population growth rate in the geographical space suggesting a significantly higher relative population growth rate at the Southern Central Andes and Northern South Andes during earlier times of the colonization process (from 15,100 to 13,500 cal BP, and from 13,500 to 11,500 cal BP breaks). Archaeological evidence is extremely sparse or absent in other areas of South America (Fig 5). This may reflect actual gaps or very low density concentrations of early occupation, but it may also reflect biased allocation of research effort (e.g. Brazilian northwest, the Gran Chaco, and north-eastern South America, the Guianas and Suriname, where archaeological research on early periods has not yet been systematically undertaken), and the visibility or level of preservation of the archaeological record. Conversely, in the case of Patagonia higher rate population growth could be related to oversampling compared with the rest of the continent. From ~11,000 cal BP onward most of the regions of South America had been fully occupied (Fig 5).

**Fig 7:**
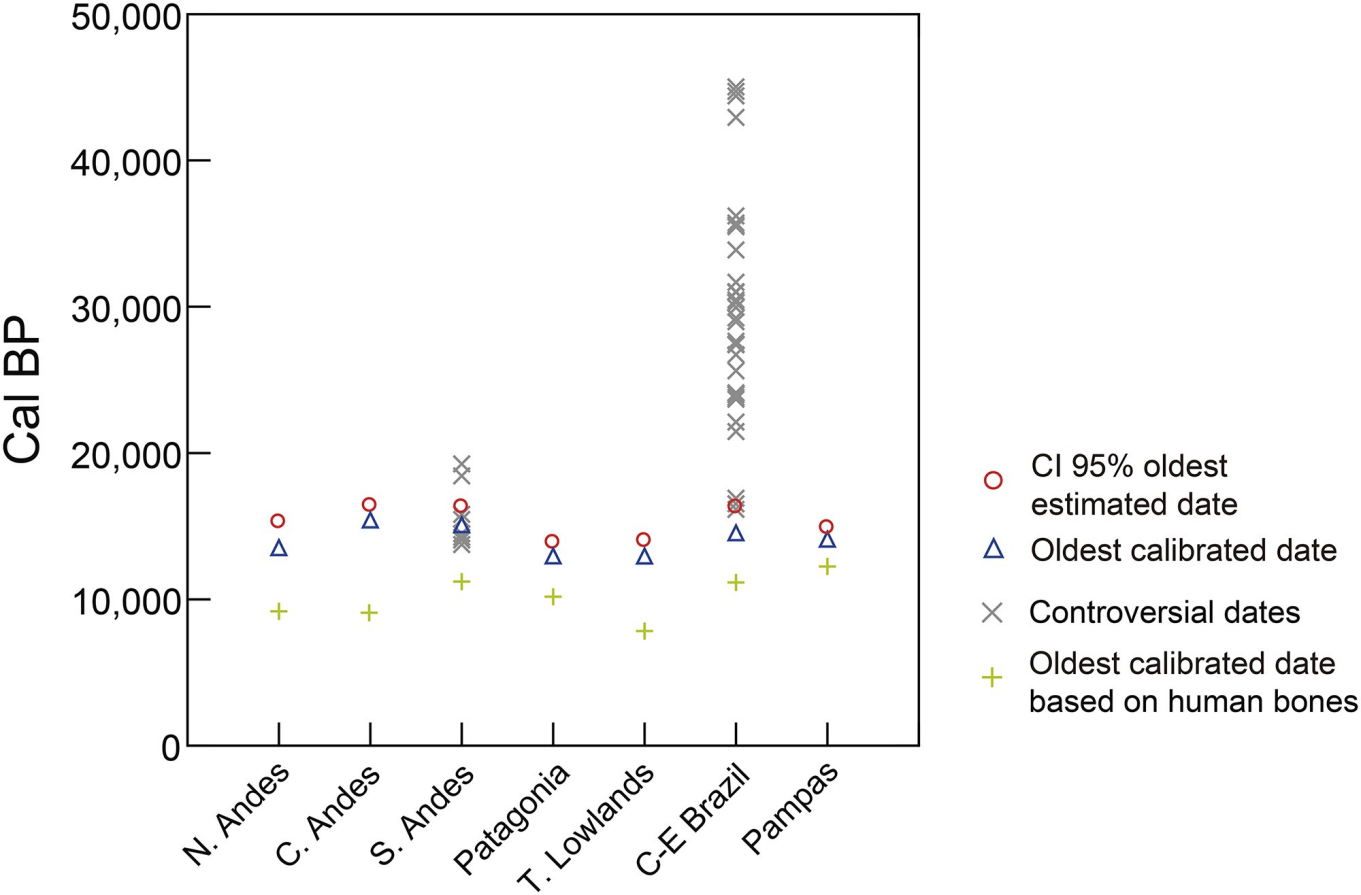
Comparison between the oldest dates, and the oldest human remains in each of the regions, and the pre-20,000 cal BP controversial dates (from Pedra Furada, Vale a Pedra Furada).

Similarly to the archaeological pattern taken as a whole, the oldest human burials from South America are numerically sparse and geographically dispersed (see Fig 1). These remains, with ages ranging from 12,550 to 12,030 cal BP, come from well dated sites in southern regions: Pampas, and South Andes [48]. The time period between the ages of the oldest calibrated date and the oldest date on human remain range from ca. 1,700 years in Pampas to ca. 6,000 in Central Andes (Fig 7).

**Fig 8:**
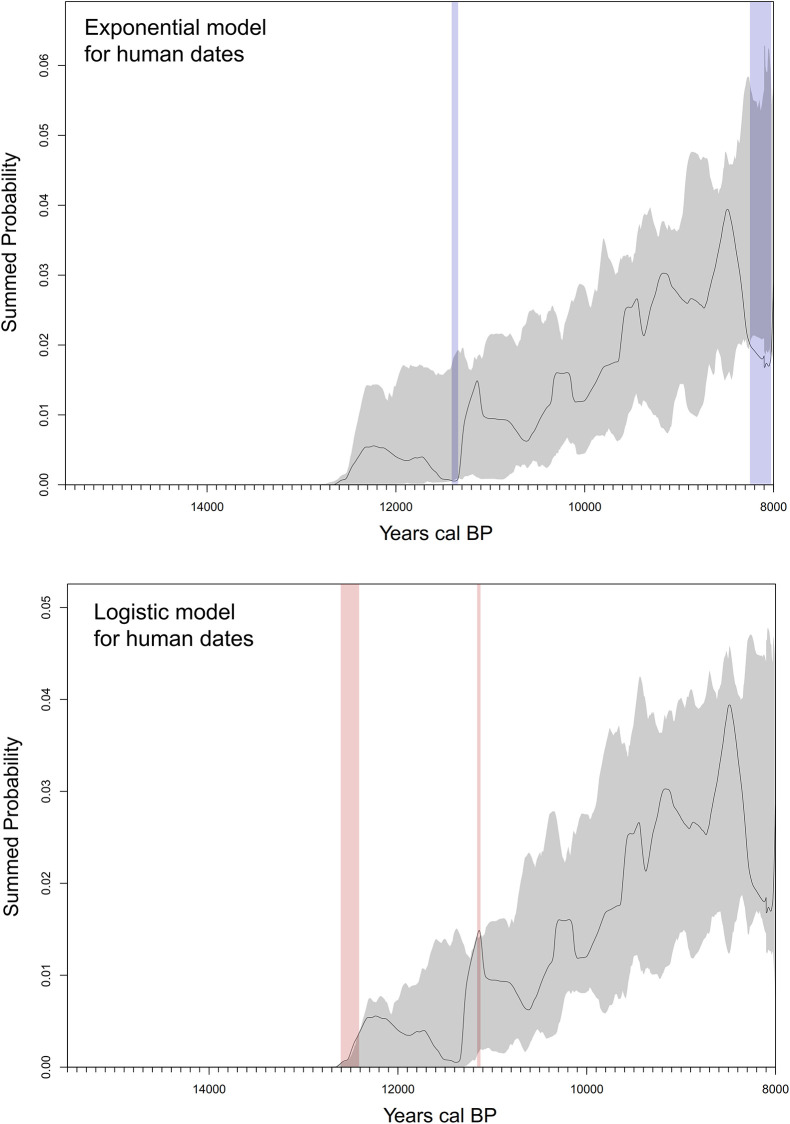
Summed probability distributions and Model Fit for human remains radiocarbon dates. ^14^C Dates (black line), with fitted null model and its 95% confidence interval (grey-shaded area) for an exponential fit (top) and logistic fit (bottom). Light red-shaded areas indicate regions that are above the expectation model’s confidence interval, whereas light blue shading indicates regions below. Figure generated with rcarbon package for R [39].

When exploring the temporal changes in only dates from human burial, it is observed that during the first 1,200 years the SCPD curve is very low and discontinuous (Fig 8). Dates for this period come from diverse regions (South Andes, Central Andes, Central/Eastern Brazil, Pampas), but only after 10,300 cal BP is there a geographically wide-ranging record of human remains (n = 105), including most regions (especially Central/Eastern Brazil, South Andes, Pampas, and Patagonia). Although from this time to the younger end of the database’s temporal range some peaks and troughs can be observed in the SCPD curve, these do not indicate any long-term departure from trend. Thus, the human remains record behaves in a similar way to the overall archaeological one during the first millennia of observed occupation, namely, after an initial period with sparse signal, a rapid logistic increase in visibility and density (Table 1). However, when we compared both curves using a permutation test, some obvious differences can be observed during the first millennium and relatively higher densities after 9000 cal BP (see S4 Fig).

## Discussion

### Timing of the human arrival to South America

Clear archaeological evidence appears in South America between ~15,100 and 13,000 cal BP. These first signals are sparse, quantitatively scarce, but clear in a few well-dated sites such as Huaca Prieta, Monte Verde II, and Arroyo Seco 2, and are probably unlikely to represent the actual colonization time. Though many scholars have indirectly considered the time when the ice-free corridor (or Pacific Rim coastal) became geographically passable as a chronological threshold for the first migration into America, most estimations of first arrival were based on independent genetic variation. Several recent genetic studies have proposed an initial entry of people south of the Laurentide ice sheet between *ca*. 19,500–14,000 cal BP [16, 19, 21], and for humans to have reached southern South America between 18,500 and 15,000 cal BP [17, 22]. Most of these time intervals based on molecular data are in a general agreement with the results of this paper, which suggests the time for the first human arrival into northern South America to have occurred sometime between 16,600–15,100 cal BP (Fig 2), and probably at 15,500 cal BP.

Our results also agree with the observed cultural heterogeneity of the earliest South American sites. Huaca Prieta, located in the Central Andean region, has been interpreted as associated with an economy focused primarily on resources from the seashore, marshes, and coastal plains [49]; Monte Verde II, located in the South Andes (in a cool, temperate rainforest), has been attributed to semi-sedentary, economically generalist hunter-gatherers [50]; and Arroyo Seco 2, located in the Southern Pampas (in a temperate grassland) was interpreted as a megamammal processing field camp within a high mobility pattern [51]. We assume the cultural homogeneity of the very first people who entered into South America based on a) the geography of the itmus of Panama, a narrow corridor that would have produced a bottleneck effect and limited the amount and diversity of people advancing south at the same time into a completely new environment, and b) recent ancient wide-genome evidences suggest that the people who arrived into South America derived from a small and genetically homogeneous group of people [16]. In consequence, the technological, economic and adaptive diversity reflected in the archaeological record of such widespread sites (Monte Verde II, Huaca Prieta and Arroyo Seco 2) can be only explained considering a greater chronological depth to human presence than is indicated by the oldest archaeological signal [52]. The estimated time for the first arrival provides a reasonable chronological framework for understanding such differences. Although a rapid increase of the archaeological visibility cannot be dismissed during colonization dispersals, the weak archaeological signal observed during the first period of the peopling is a reasonable correlate of human dispersals into previously uninhabited areas [23, 26, 53].

A range between 16,600 and 15,100 cal BP, with a mean date of 15,500 cal BP, as the most probable time of first human entrance into South America is also congruent with the direct dates on human skeletal remains. Burials known from the late Pleistocene of South America are extremely scarce in contrast to their more visible presence in other parts of the world [5]. The time period separating the oldest cultural and the oldest human skeletal remains varies from ~1,800 years (in Pampas and Patagonia) to ~5,000 (in Central Andes). Some scholars have previously considered the paucity or delayed appearance of human remains–compared with material culture–in the Americas as an anomaly needed to be explained. This has been interpreted as the result of multiple potential causes (natural, cultural and scientific): lack of understanding of mortuary practices for this period, archaeological sampling biases, research trajectories and effort, social organization, and death-related behaviors–for example high frequencies of body abandonment–, facilitated by socioecological factors–absence of spatial circumscription–[54]. It is not the aim here to provide in-depth evaluation of those hypotheses or deny the many determining factors of such an empirical trend, but our results do not necessarily entail any evident anomaly. The time separating the oldest cultural and the oldest human skeletal remains varies quite similarly over regions, with an average value of ~2,800 (Fig 7). Although this time lag does not have predictive value, the material culture is usually much more abundant than human skeletons in the archaeological record, since individuals generate a significantly higher amount of material waste (e.g., lithics, faunal remains, charcoal, etc.) during their lifetime compared with their own bone remains. Therefore, evidence for the first human presence could be expected to come from material culture first, and for the oldest skeletal remains to be more recent. It is also expected for burials (or even human bones naturally embedded in the stratigraphy as any other organism) to become archaeologically visible after humans have reached some threshold of population density. The results of this paper support this expectation since some congruence between the first human remains and an increase in the archaeological signal is clearly noticeable when comparing the corresponding SCPD curves (see S4 Fig). The threshold of the first amplification of the cultural signal matches in time with the first human remains (~12,500 cal BP), and the first amplification of the human remains signal also matches with a second (and weak) amplification of the cultural signal.

The results of this paper suggest that humans entered South America between 16,600 and 15,100 cal BP, which is in disagreement with several claims of a high antiquity entry based on presumed long chronology sites in Brazil [11] and Uruguay [55]. Beyond some of the unsolved ambiguities that most of these sites still present (see SI Text), SCPD curve generated with the non-conservative dataset–which includes the excluded dates–(S1 Fig) does not behave as expected in the human colonization of an inhabited area (see curves of demographic population growth in 21, 24]; mainly because of the extremely long period of intermittent, low and discontinuous archaeological signal (S1 Fig *-top* and S5 Fig. Moreover, the chronological discontinuity observed between these ages and oldest South American human skeletons becomes difficult to explain by assuming a continuous human occupation of the continent. If these early dates were actual evidence of human presence in South America, a strong explanation for such severe inconsistences must be provided. It seems more reasonable to us that these pre-LGM sites were not associated with human origin, which makes sense in the light of several recent arguments which call into question the human agency in the Piauí region sites (Brazil) [13, 56, 57].

In the same way that this paper does not support an initial human occupation of South America before 16,600 cal BP, our estimated date for the first arrival calls into question some of the expectations drawn from the Short Chronology Model. On one hand, it is not known how quickly foragers dispersed from Alaska to Panamá, but assuming that Clovis sites represent a demic human expansion with a high rate of dispersals and rapid population growth rates, this process must have lasted ca. 2000 year, as estimated by previous dispersal models [23, 26]. So, if humans reached northern South America between 16,600 and 15,100 cal BP, they must have entered North America not later than 17,400 cal BP. Moreover, if we consider a period of around 1500 years of invisibility of Clovis (between 14,400 and 12,900 cal BP) due to taphonomic and sampling effects [24], a discrepancy of ca. 1000 years emerges between expectations for North America and South America. It is likely that this discrepancy could only be explained by a pre-Clovis occupation east of North America's Rocky Mountains and/or an initial dispersal mainly Southward through a Pacific Ocean Coast Corridor, which has not been empirically supported as of yet, as Surovell has clearly noted many years ago [58]. The latter would necessarily imply “a dispersal process with low equilibrium fitness where scattered humans were expanding through the continent(s) not reaching the minimum viable population density necessary to produce a completely-filled-environment human dispersal” [26]. On the other hand, considering our conservative dataset (which does not include pre-Clovis dates), density maps of radiocarbon dates (Fig 5b) show that the archaeological signal reaches most of the region of South America almost simultaneously during the first centuries with human bones, and the SCPD curve (S1 Fig *-bottom*) reveals a positive steep suddenly after the archaeological signal appears. Although an alternative scenario of quick peopling does not fully agree with what’s expected in a colonization process [23, 26], it cannot be rejected completely in case of extremely high rates of dispersal and exploratory mobility [59].

### Demographic growth and spatial dispersion of early human populations

After a first period of low-density of archaeological signal in South America (between ~15,100 and 13,000 cal BP), chronological data shows a clear and rapid amplification at ~13,000 cal BP, which fits well with estimated by simulated dispersal models [23, 26] that predict a variable period of low population density before human dispersal produces a completely-filled-environment. It is at that time that the sites in the region associated primarily with fishtail projectile points (e.g. Je 996, Je 1002, Fells Cave, Salar de Punta Negra 1, Abrigo Los Pinos, Cerro La China I, Paso Otero 5, Piedra Museo, Jaywamachay, El Palto, Tagua Tagua 2, etc.) and secondarily with other types of projectile points such as Tuina, Paiján and El Tigre [9, 60] appeared. This has two main implications for discussing the peopling process. First, that by the time the Clovis culture spread throughout North America, human beings had been living in South America long enough to leave a widely visible and diverse archaeological signal, and probably developed other regional projectile point traditions not related to Clovis [61]. And second, that fishtail projectile points (some of them fluted) appear in South America significantly later–perhaps as much as 500–2000 years later depending on location–than the oldest archaeological evidence.

From a demographic perspective, the SCPD curve data reveals a long-term trend that behaves like a logistic curve. This is at variance with the growth curves generated using similar methods for North America and Europe [35], but this generally agrees with Goldberg *et al*.’s South American analysis [7], even though they used an unscreened database. These authors fitted a logistic curve for the whole period, from first colonization to around 5500 cal BP, followed by exponential growth onwards probably associated with widespread sedentism. The logistic growth is characterized by an initial rapid growth, followed by a slowing-down as the carrying capacity, established by the available resources, is approached. It has been observed in many different species [62] and, according to Goldberg *et al*. [7], it is characteristic of populations migrating into favorable habitats. Beyond the best fit of the logistic model, there are two main spikes that take the SCPD curve. The oldest is observed around 12,500 cal BP and it is suggestive that it fits well chronologically with the end of Antarctic Cold Reversal (ACR) stadial. After that period, not only important ecological and climate changes occurred in South America [63], but most of megafauna families became extinct, including those exploited by humans (Mylodontinae, Equidae, Megatheriidaee, Glyptodontidae and Gomphoteriidae) [64]. Such a significant change occurred in the trophic niche of humans could have induced (or enhanced) the slow of the initial and rapid population growth. The latest spikes of the SCPD curve occur at ~8400 years cal BP and seem to fit well chronologically with the widespread appearance of specialized coastal economies in South America which could have also induced a new rapid population growth. If these two spikes reflect irruptive growth dynamics with overshoot of long-term carrying capacity and not an over-representation of early sites–in the former case–, and of shellmiddens in post glacial times–in the latter–a stepped population curve characterized by several successive logistic models, more than a simple’s logistic model, should be evaluated in the future.

Regarding the spatial distribution of radiocarbon dates (Figs 5 and 6, and S3 Fig), no gradient suggesting directionality in the peopling process is observed. Rather, the clearest trend emerging from the density map (Fig 5) is that the earliest dates are concentrated in a few regions, especially in Central Andes, South Andes and Pampas. That general trend of the spatial distribution of dates, with a high concentration of early southern sites, seems to be incompatible with the predictions of a north-to-south expansion pattern (i.e. a decreasing age of the oldest evidence from north to south). Although that picture could be affected by taphonomic effects and by an uneven coverage of research–for example a provable over-representation of Patagonia and under-representation of Chaco and Amazonas [6]–, it seems congruent with: a) a rapid human dispersal from North America through coastal corridors and a secondary migration to Pampas; b) the higher relative population growth rate estimated in the Southern Central Andes and Northern South Andes (compared with other regions) between 15,100 and 11,500 cal BP (Fig 6 and S3 Fig); c) previous expectation, based on continental scale elevation data and GIS analyses, about the least-cost route for South American earliest human dispersion [58]; and d) recent genetic-based studies which use variation of Mitochondrial DNA haplogroup to derive two migration routes, the main in the western, along the Andes or Pacific coast Andes, and probably another in the eastern along lowlands or Atlantic coast [8, 17]. If this was the case, one could expect a shorter chronological difference between oldest dates in North and South America [26].

## Conclusions

In this paper we have offered possible answers for longstanding controversies on the peopling of South America using radiocarbon dates and a quantitative approach. We have estimated that the earliest chronological threshold for the peopling of South America should be between 15,100 and 16,600 cal BP, (mean date 15,500 cal BP) which implies a period between 300 and 1500 years of poorly visible or invisible archaeological signal. If a pre-15,500 cal BP first arrival indeed occurred, this early population would probably have become extinct, since alleged cultural evidence before this date shows a substantial (and unexpected) discontinuity in the SCPD curve, and human remains are completely absent before 12,600 cal BP. On the other hand, our results are not congruent with expectation from the Short Chronology Model for the peopling of the Americas, which assumes that specialized hunters of megafauna (Clovis-related people) entered South America and spread southward at *ca*. 12,900 cal BP) [10]. Unlike the Long Chronology Hypothesis, which is not even supported by the curve run with the liberal (non-conservative) dataset, the Short Chronology Model cannot be completely rejected with the conservative curve by itself, but it would require the assumption of a very rapid migration, and extremely high (and unlikely) rates of initial dispersal for humans to reach the southern tip of South America (see estimates by Steele et al. [23] and Lanata et al. [26]). Moreover, the Short Chronology model does not seem congruent with reliable pre-Clovis sites from South America, and with the technological and economic diversity in the archaeological record of the southern continent by ca. 12,900 cal BP. The results of this paper are in agreement with predictions of an intermediate (pre-Clovis and post LGM) antiquity peopling of the Americas. This model necessarily supports a pre-Clovis occupation of North America [65, 66], and fits well within the range of estimates based on genetic analysis [16, 17, 19, 21, 22].

After humans dispersed into the Southern Continent, they seem to have occupied, relatively rapidly and with low population density, the main regions of South America at around 13,000 cal BP, from the Central Andes and North Eastern Brazil to Patagonia, and used a variety of ecological niches and different types of technology. Then, population would have grown until the end of Antarctic Cold Reversal stadial– ~12,500 cal BP at the time of the main extinctions of megafauna–, when the increase rate slows, probably as a result of the changes occurred in the trophic niche of humans. By the time of this population growth, new technology associated with the fishtail projectile points (and a few other projectile point types) along with other potential cultural innovations (for example new hunting strategies, more efficient use of the raw material), probably generated the change or expansion of the human trophic niche, allowing for the population increase following a logistic growth model. Future studies are necessary for a deeper understanding of the cultural factors (e.g. technological innovations) behind the complex demographic history of the early peopling of South America until the population reached demographic stability at ~11,000 cal BP.

## Supporting information

S1 Fig Summed probability distributions of radiocarbon dates based on databases built with different validation criteria. Non-conservative (top), standard (middle), and conservative (bottom).
(TIF)

S2 Fig Summed probability distributions of radiocarbon of different datasets of radiocarbon dates. The different datasets include whole dates and sets with different filtering of dates (removing shell/charcoal/single dates, and with different values of error). The SPD curves of 14C dates are shown as black line, with permutation 95% confidence interval in grey-shaded area. Red-shaded areas indicate regions that are above the permutation confidence interval, whereas blue shading indicates regions below. Figure generated with rcarbon package for R (Bevan A, Crema ER (2018). *rcarbon*: *Methods for calibrating and analysing radiocarbon dates*. https://github.com/ahb108/rcarbon.).
(TIF)

S3 Fig Local and global geometric growth rate in South America in three transitions. The three breaks and four intervals of 2,000 years were estimated on the SCPD curve with the rcarbon package for R (Bevan A, Crema ER (2018). *rcarbon*: *Methods for calibrating and analysing radiocarbon dates*. https://github.com/ahb108/rcarbon.). The upper part of the figure shows the local geometric growth rate on a map of South America, whereas the lower portion displays the global growth rate for the subcontinent.
(TIF)

S4 Fig Summed probability distributions of radiocarbon human dates versus all other dates. 14C Dates (black line), with permutation 95% confidence interval (grey-shaded area). Red-shaded areas indicate regions that are above the permutation confidence interval, whereas blue shading indicates regions below. Figure generated with rcarbon package for R (Bevan A, Crema ER (2018). *rcarbon*: *Methods for calibrating and analysing radiocarbon dates*. https://github.com/ahb108/rcarbon.).
(TIF)

S5 Fig Oldest estimated date of first human arrival compared with dates from controversial sites. (TIF)

S1 Table Time periods of first arrival in different regions of South America estimated by using different methods. (DOCX)

S2 Table Time periods of first arrival in whole South America estimated by using different methods and different filtering of dates. Dates from multi-dated sites only (left), dates from multi-dated sites excluding charcoal, and dates from multi-dated sites excluding charcoal and shell (right).
(DOCX)

S1 Dataset Dates included in the analysis. For each of the radiocarbon dates, the following information was included: site name, region, ^14^C age, laboratory code, and full reference. Oldest reliable radiocarbon date of each region is highlighted in red.
(XLSX)

S2 Dataset Dates excluded from the analysis because these do not fit our validity criteria. For each of the radiocarbon dates, the following information was included: site name, region, ^14^C age, laboratory code, and full reference.
(XLSX)

S1 Text Grounds for excluding from the analysis the pre-15,000 BP dates from several renowned South American sites. Toca do Boquerao da Pedra Furada, Vale da Pedra Furada, Toca do Sitio do Meio, Toca da Tira Peia; and Chinchihuapi / Monte.
(DOCX)
