# Supplementary figures and images for: Rapid radiation of humans in South America after the last glacial maximum: A radiocarbon-based study
Source: PLoS One. 2020 Jul 22;15(7):e0236023. doi: 10.1371/journal.pone.0236023 (PMC7375534; doi:10.1371/journal.pone.0236023)

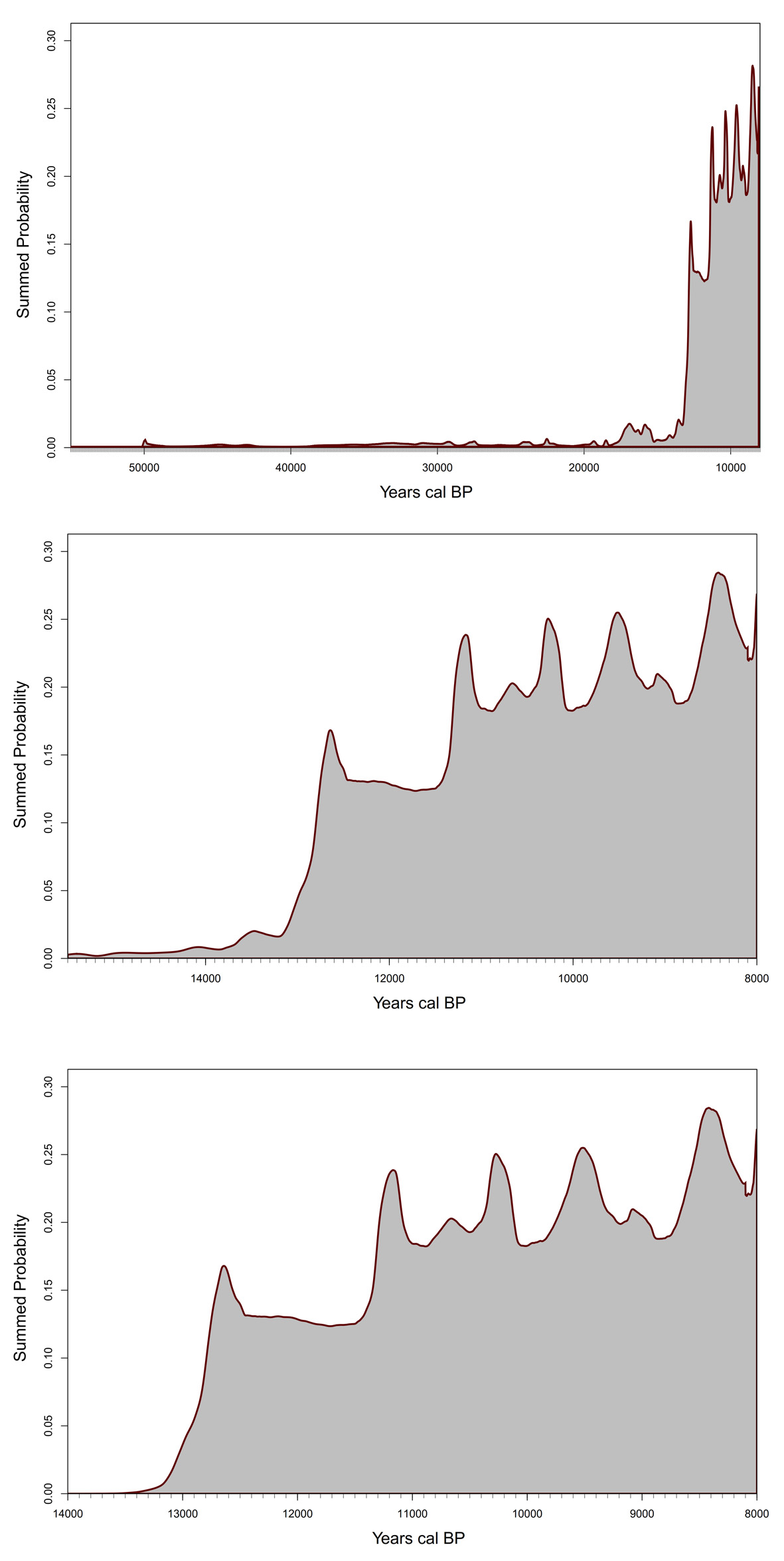

Supplement: S1 Fig — Non-conservative (top), standard (middle), and conservative (bottom). (TIF) [file pone.0236023.s001.tif]

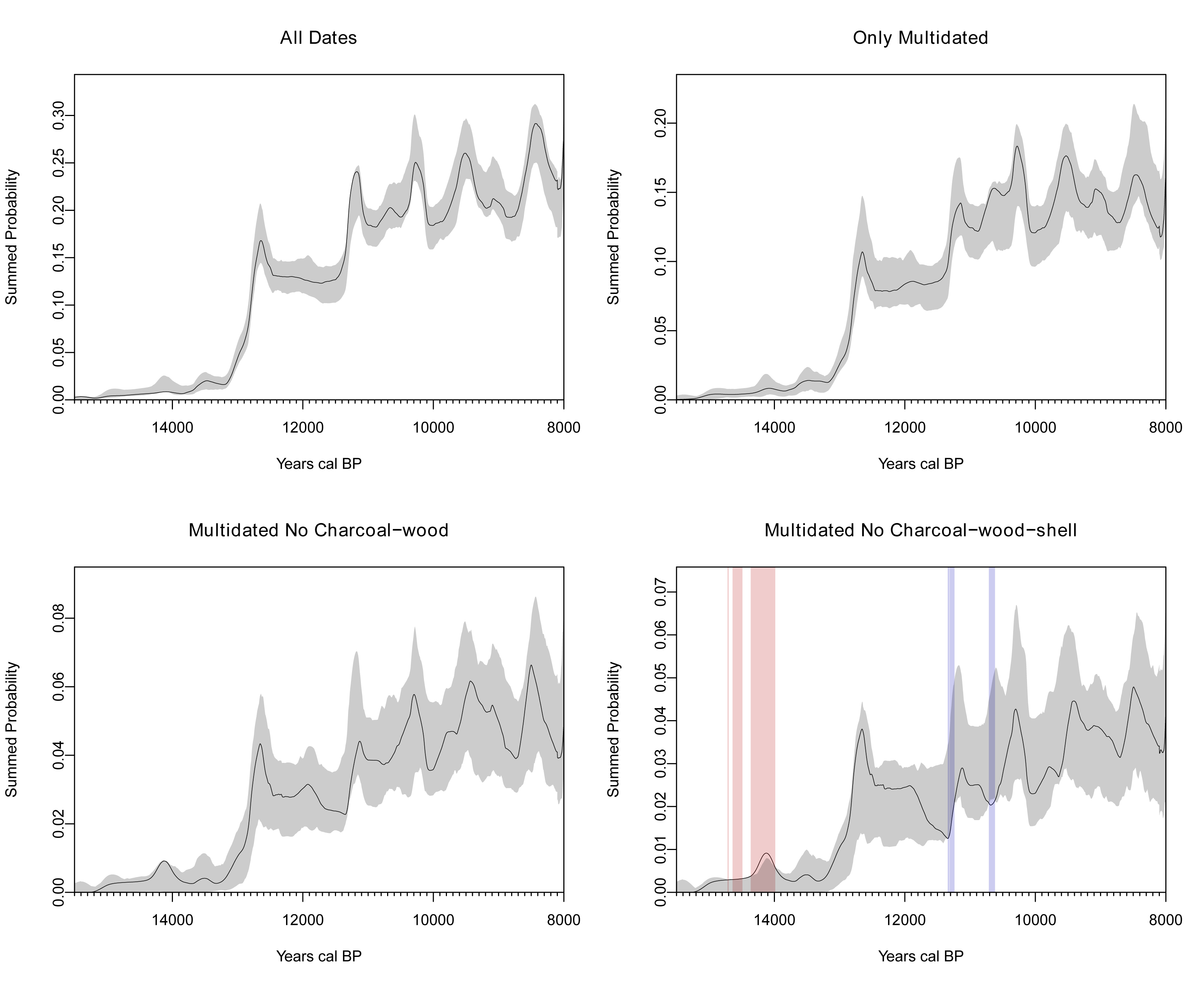

Supplement: S2 Fig — The different datasets include whole dates and sets with different filtering of dates (removing shell/charcoal/single dates, and with different values of error). The SPD curves of 14C dates are shown as black line, with permutation 95% confidence interval in grey-shaded area. Red-shaded areas indicate regions that are above the permutation confidence interval, whereas blue shading indicates regions below. Figure generated with rcarbon package for R (Bevan A, Crema ER (2018). rcarbon: Methods for calibrating and analysing radiocarbon dates. https://github.com/ahb108/rcarbon.). (TIF) [file pone.0236023.s002.tif]

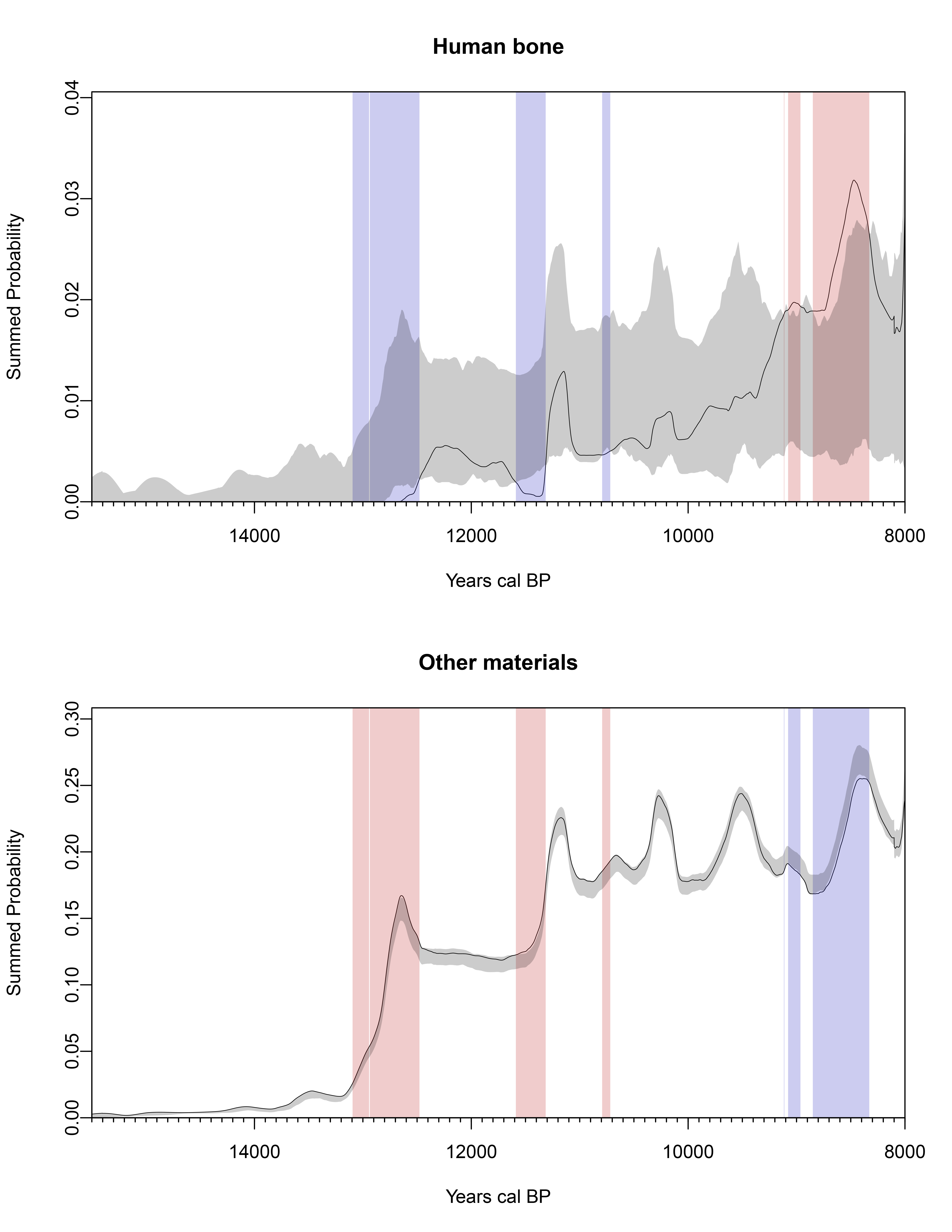

Supplement: S4 Fig — 14C Dates (black line), with permutation 95% confidence interval (grey-shaded area). Red-shaded areas indicate regions that are above the permutation confidence interval, whereas blue shading indicates regions below. Figure generated with rcarbon package for R (Bevan A, Crema ER (2018). rcarbon: Methods for calibrating and analysing radiocarbon dates. https://github.com/ahb108/rcarbon.). (TIF) [file pone.0236023.s004.tif]

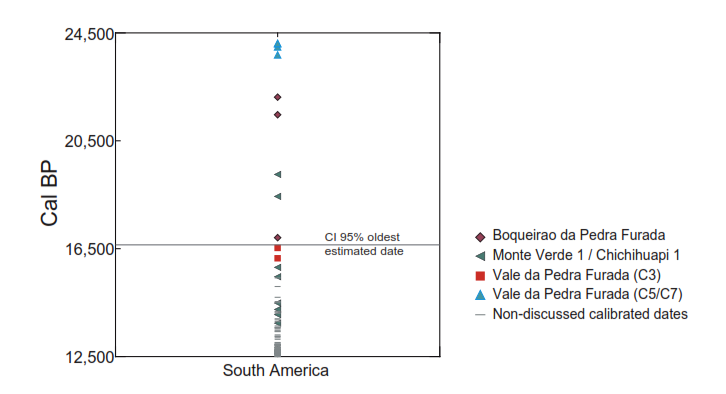

Supplement: S5 Fig — (TIF) [file pone.0236023.s005.tif]
