# Supplementary material for: Rapid radiation of humans in South America after the last glacial maximum: A radiocarbon-based study
Source: PLoS One. 2020 Jul 22;15(7):e0236023. doi: 10.1371/journal.pone.0236023 (PMC7375534; doi:10.1371/journal.pone.0236023)
Supplement: S1 Table — (DOCX) [file pone.0236023.s006.docx]

Table S1. Time periods of first arrival to different regions and whole South America estimated by using different methods.

|  | **North Andes** | **Central Andes** | **South Andes** | **Patagonia** | **Tropical Lowlands** | **Central East Brazil** | **Pampas** |
| --- | --- | --- | --- | --- | --- | --- | --- |
| **Strauss_Sadler** | [12780 - 12960] | [14907 - 14979] | [15096 - 15173] | [12962 - 13049] | [12958 - 13176] | [13903 - 13974] | [14090 - 14241] |
| **Solow** | [12777 - 12931] | [14906 - 14982] | [15095 - 15148] | [12961 - 13063] | [12954 - 13195] | [13902 - 13957] | [14087 - 14260] |
| **Marshall** | [12777 - 13677] | [14906 - 16065] | [15095 - 17336] | [12961 - 14309] | [12954 - 14368] | [13904 - 16164] | [14087 - 14828] |
| **McCarthy** | [12778 - 12785] | [14907 - 14945] | [15096 - 15134] | [12962 - 12965] | [12955 - 12967] | [13904 - 13965] | [14088 - 14098] |
| **McInerny** | [12323 - 12769] | [14755 - 14903] | [14936 - 15092] | [12744 - 12957] | [12417 - 12945] | [13741 - 13899] | [13751 - 14081] |
| **Griwm** | [13003 - 13008] | [15055 - 15056] | [15296 - 15298] | [13166 - 13168] | [13291 - 13295] | [14217 - 14222] | [14265 - 14266] |

Highlighted in gray the used methods in this paper
