# Supplementary material for: Rapid radiation of humans in South America after the last glacial maximum: A radiocarbon-based study
Source: PLoS One. 2020 Jul 22;15(7):e0236023. doi: 10.1371/journal.pone.0236023 (PMC7375534; doi:10.1371/journal.pone.0236023)
Supplement: S2 Table — Dates from multi-dated sites only (left), dates from multi-dated sites excluding charcoal, and dates from multi-dated sites excluding charcoal and shell (right). (DOCX) [file pone.0236023.s007.docx]

Table S2. Time periods of first arrival in whole South America estimated by using different methods and different filtering of dates: dates from multi-dated sites only (left), dates from multi-dated sites excluding charcoal, and dates from multi-dated sites excluding charcoal and shell (right)

|  |  |  |  |
| --- | --- | --- | --- |
|  | **Multi-dated** | **Multi-dated (without charcoal/wood)** | **Multi-dated (without charcoal/wood/shell)** |
| **Strauss_Sadler** | [15095 - 15112] | [14851 - 14900] | [14851 - 14914] |
| **Solow** | [15095 - 15099] | [14850 - 14890] | [14850 - 14899] |
| **Marshall** | **[15107 - 16612]** | **[14850 - 16075]** | **[14850 - 16276]** |
| **McCarthy** | [15097 - 15230] | [14851 - 14903] | [14851 - 14891] |
| **McInerny** | [15061 - 15094] | [14747 - 14848] | [14717 - 14848] |
| **Griwm** | [15167 - 15172] | [14979 - 14980] | [14998 - 14999] |

Highlighted in gray the used method in this paper
