# Supplementary material for: Rapid radiation of humans in South America after the last glacial maximum: A radiocarbon-based study
Source: PLoS One. 2020 Jul 22;15(7):e0236023. doi: 10.1371/journal.pone.0236023 (PMC7375534; doi:10.1371/journal.pone.0236023)
Supplement: S1 Text — Toca do Boquerao da Pedra Furada, Vale da Pedra Furada, Toca do Sitio do Meio, Toca da Tira Peia; and Chinchihuapi / Monte. (DOCX) [file pone.0236023.s010.docx]

**Grounds for excluding dates from controversial South American sites from the analysis**

Although discussing individual sites is far beyond the aims of the paper, in the following lines we briefly refer to the criteria for excluding several multi-dated (and widely known) but somehow controversial early South American sites (Toca do Boquerao da Pedra Furada (BPF) [1], Vale da Pedra Furada (VPF) [2], Toca do Sitio do Meio (TSM) [3], Toca da Tira Peia (TTP) [4]; Santa Elina (SE) [5], Pilauco (PI) [6], Chinchihuapi I (pre-14,500 cal BP levels and II / Monte Verde 1 (CH/MVI) [7, 8], and Arroyo Vizcaíno [9]), and b) two sites with early human remains (Lapa Vermelha and Toca do Gordo do Garrincho [10, 11]). We are not rejecting, dropping or invalidating these sites. We are simply applying some criteria to include their 14C dates as a proxies for human occupation in order to construct a data base to perform the statistical analysis developed in this paper. Of course, the inclusion/exclusion of dates have implications in the results we are obtaining and we are aware about this, but by no mean we are proposing that these sites should be ruled out of the discussion about the peopling of he America. Their exclusion derive from the criteria and the methodology we applied and are guided by specific goals.

*Serra da Capivara sites (BPF, VPF, TSM, TTP)*

Research into all presumed pre-15,000 BP sites from Brazil has been carried out by international archaeological missions, involving basically the participation of French and Brazilian archaeologists. In Serra da Capivara region, in Southern Piauí State, the best known and published site is BPF, a huge sandstone rockshelter in the southeastern. Since the 1980 decade, Guidon and his team have been publishing several papers with partial data about the site [i.e. 12] until the extensive monograph of Parenti [13] that serves as a final report. The archaeological sequence of the site has been dated between 5,000 to more than 60,000 yrs BP [14], although dates up to 100,000 yrs BP have also been suggested. The site was object to criticism [15] and rebuttals [1] which clarified part of the problems. A balanced and contemporary perspective from the side of the defenders can be seen in Parenti [14] who states that “today its [BPF] informative potential is largely underestimated, both because of its uniqueness in South American archaeological context, but also because of the intrinsic nature of the debate on antiquity of peopling, with an exhausting succession of announcements and dismissals, and the lack of fully Pleistocenic human remains in Americas.” (pp. 5844). The main points of contention are the human origin of the stone tools and of the hearths. No animal or human bones have been recovered in the site. One of the main problems of the site is that all the artifacts are made on local quartz and quartzite which outcrops in the roof the rock shelter which produced a “cooble rain” fell for tens of thousands of years in the site; the possibility of the creation of pseudo-artifacts cannot be ruled out (but see [13] for a discussion about how to discriminate them) and the vertical migration of artifacts from the upper Holocene layers (the Serra Talhada Phase) is always a potential source for a secondary association. The recent reports of human-like artefacts made by Capuchins monkey in the Serra da Capivara region [16, 17] introduce a new technological agent which has to be considered in the discussion. Beside of the ambiguous character of some of the stone tools and of the hearts, the main problem as Parenti [13] states is that “ Pedra Furada has not yet definitely and fully been accepted in archaeological literature, and it will probably remain in this limbo situation until the discovery of robust and dated palaeoanthropological evidence in the region” (pp, 5053).

Based on the study of new sites in Serra da Capivara Boëda and his team present an occupation sequence that covers more than 20,000 years, from ca. 35,000 to ca. 12,000 cal BP [18]. None of these four sites showed a continuous occupation, and the presumptive archaeological layers are separated by thousands of years (such is the case in VPF, TTP and SM). In an attempt to articulate the data obtained for BPF, VPF and TSM, the authors proposed three main periods of occupation in this region during the Late Pleistocene, two of them before the LGM and the last one after the LGM: the first period between 35,000 and 27/28,000 BP, when the area is asserted to have been occupied regularly; the second starts around 24,000 and last for a few thousand years; and the third ranges from 16/17,000 BP into the early Holocene [4]. Despite the enormous impact that this scenario, if proven, would have on understanding of the early peopling of the Americas, most of the more recent papers are subject to the same unresolved issues as those brought to light in a debate during the mid-1990s [14, 15]. The main issue is the lack of a detailed taphonomic study for each site, including cultural and natural post-depositional processes.

Another crucial issue regarding these sites is the lack of quantitative data relating to the lithic assemblages and their spatial distribution within and between archaeological layers. The emphasis is made on “proving” the human origin of these artefacts (using principles whose universality has been called into question in a recent paper [19], without published data on how many tools, cores, and flakes were found and how many ecofacts were present in the matrix. For Vale da Pedra Furada, for example, considering the long history of research into the site, it would be welcome to know more about the stratigraphic distribution and technological features of the lithics. A similar problem lies with Toca da Tira Peia, not only regarding lithic material but also regarding charcoal, as it is not clear if the latter samples came from structured hearths, spatial concentrations, or if they were randomly collected from dispersed charcoal [20], which would not securely be from human origin. But perhaps the most important problematic issue with these early sites from Piauí region (other than the potential non-human origin of some lithic assemblages [16, 17] is the very limited published information about the spatial and cultural association between lithic remains and charcoal, which would imply a weak association between putative human event (the lithics) and dated charcoal sample. In sum, for the claims made to be more widely accepted it would be necessary to recognize the burden of proof that rests with the excavators when asserting a claim to have discovered a very early site. Work has already been undertaken to construct a robust chronology using different dating methods, and to present a detailed technological analysis of the artefacts found in these sites. Certainly, with a similar effort put into taphonomic analysis to better understand the site formation processes, much of the uncertainty related to these sites will be resolved. This would undoubtedly contribute to clarifying and possibly reframing the debate about early peopling of South America.

*Santa Elina*

The Santa Elina shelter is placed in the Serra Mountain in the State of Mato Grosso, Brazil. Fieldworks were done between 1984 and 2004 by Denis and Agueda Vialou and included the excavation of an area of 80 m^2^ [21]. The authors identified two periods of human occupation, with an age of 23,120±260 BP of the first, and of 10,120±60 BP of the second. While the excavation has been very carefully addressed and there is a monograph with all the information about the site [22], we did not include the oldest set of dates in our analysis due to two main reasons: a) the age of the oldest occupational layer was defined based on three dates: 23,320±1000 BP (Gif-9365; 25,519 – 29,488 cal BP), 22,500±500 BP (Gif-9366; 25,833 – 27603cal BP), and 23,120±260 BP (GIFA-99177; 27,818 – 26,887 cal BP). The first two dates (obtained from samples of naturally saturated wood from a waterlogged deposit) were obtained from OSL and U/Th dating method, which are beyond the scope of this paper. They also produced greater sigma of those that we accept in the previously exposed validation criteria. b) The only ^14^C date of the oldest unit (23,120±260 BP) was obtained from microcharcoals from a thin and ashy layer. Applying the criteria mentioned above. Since these microcharcoals are not clearly associated to a human-made feature (such as a hearth) they might not be dating human activity.

*Monte Verde I / Chinchihuapi I and II*

Archaeological research in Chinchihuapi Creek (Central Chile), have yield several final Pleistocene deposits (MV 1; MV2, Chinchihuapi,1 and Chinchihuapi 2), but until recently MV2, with an age of ca. 14.5 kyr cal BP, had been the only one systematically studied and published [23]. Nevertheless, new research into MV1, and Chinchihuapi I and II, has suggested that humans may have settled in the area 4,000 / 10,000 years before previously thought, i.e. between 14.5 and 19/25 kyr BP [7]. Although the new findings are suggestive, we consider that the available evidence is still weak to strongly support this hypothesis (with the exception of the recently published levels dated between ~ 9500 and ~ 14,500 cal yrs BP from Chinchihuapi I, [8]). The main issue is that the density of archaeological evidence recovered in the new excavations from the sites is extremely low (on both spatial and chronological scales): 39 lithics, 12 burned features and 8 unidentifiied bone fragments from an excavated area of ca. 200 m^2^, and spanning more than 10,000 years (from ca. 10,600 to 25,000 years). Even considering the possibility that these contexts would represent ephemeral occupations, the scarcity of the materials (including some of them with a dubious status) and the horizontal and vertical discontinuity of the remains make difficult to confirm human activity earlier than ~ 14,500 cal BP. We agree with Dillehay who in a recent publication and in reference to MV I, Ch-I and II stated that “In addition, there are other lithic, wood, charcoal, and burned bones dated between ̴ 16,000 and at least 20,000 cal yrs BP, that were not found with use surface but were recovered from the lower stratified intact layers. These features and materials and sequences requires more contextual evidence and firm dating” [8:9, underlie is our]. Therefore, at its present status we decided not to include yet the pre-14,500 ^14^C dates from these sites in our data base.

*Pilauco.*

This site is located in a suburb of Osorno city, Chile. For several years the archaeological information from this site was provided mainly by one article [6; see also 24], while several others basically addressed geological and paleontological issues [e.g. 25, 26, 27, 28]. After several years, two new papers presented more date about the cultural materials (including an isolated footprint), which would make arguments stronger, however, we still consider they do not meet the validation standards set before [29, 30]. This do not diminish the potentiality of the site, but stress the point that in despite of the abundance of information presented, there are still some doubts that have to be resolved, especially regarding the radiocarbon dates, their stratigraphic position and their cultural association. For example: a) There are inconsistencies and contradictions in the presentation of the nine radiocarbon dates [6]. Only one calibrated date (12,508 BP) in Table 1 match with the values depicted in Figure 3; all the others have differences in the values in spite that they should be the same dates. In Table 1 dates are consistent with the depth, but in Figure 3 there are some inversions (12,508 is lower than 12,699, and 12,798 and 13,518 are lower than 13.698) [6]. b) There are inconsistencies in the layer location of the dated samples between papers. In Pino et al. [6] samples Geoauch -15, 53, 52, 55, 49 and 57- are placed in Layer PB-7 (Table 1, p. 6) while in Pino et al. [28] (Table S4), all these samples (except Geoauch -15) are located in the lower layer PB-8. The confusion is also present in Labarca et al. [23:258] who state that a total of 16 dates on bone, wood and charcoal “placed the Layer PB-7 between 13.220±60 (AA101831) and 11.004±186 (AA- 81812) AP uncalibrated”. However, in Pino el al. [28] (Table S4) some of these samples are located in Layer PB-8. Moreover, the former date (13.220±60; AA101831) figures in Pino et al. [28] with another lab code: UCIAMS 101831. c) Labarca et al. [29: 2] expressed that the date of 10,710 years BP (UCIAMS 101684) is placed in the top of PB-8, while in Pino et al. [28] (Table S4) it is placed in PB-9. d) Navarro Harris et al. [29:2] say that “The PB-8 and PB-9 layers are separated by a second erosional unconformity dated between 10,950±30 and 10,710±30 ^14^C year BP (Table S1)” but in Pino et al. [28] (Table S4) both dates are in PB-9. e) The lists of dates from the site presented in two articles published almost at the same time do not match. In Pino et al. [28] (Table S4) there are 27 dates in PB-7 and PB-8. In Navarro-Harris et al. [29] (Table S1) there are 38 radiocarbon dates in the same two layers but the three oldest dates from Layer PB-7 reported in Pino et al. [28] (Tables S4) are missing (dates 13,470±35; 13.570±70 and 13,650±70 BP). Navarro-Harris [29] also reported dates of 10,660±30 and 10,950±30 in Layer PB-8, while in Pino et al. [28] (Table S4) both dates are placed in Layer PB-9. On this basis, is not possible to unambiguously determine which of the 38 radiocarbon dates from layers PB-7 and PB-8 are associated with human activity.

Although the anthropic origin of some artifacts is quite clear, as well as the allochtonous source of the rhyodacitic and dacitic raw material [29] a full report of the lithic assemblage -necessary for a better understanding of the human activity at the site- is not available. Some taphonomic process identified in the site (root marks, hydric abrasion, trampling action) should be taken into account since they would produce vertical migration of the artifacts. Some evidence indicate a complex taphonomic history, and as the site investigators wrote some time ago “It is not possible to rule out the transfer and / or rearrangement of some pieces by river agents, particularly those of smaller size and rounded morphology” (our translation) [25:267]. Since most of the lithics are of small and medium size, and many of them are rounded, vertical migration in PB-7 and PB-8 is a possibility that needs to be explored in deep. Without any doubt, the research in Pilauco has a great potential but given the criteria summarized before, we decided not to include their ^14^C dates it in our data base.

*Arroyo del Vizcaíno.*

In Arroyo del Vizcaíno site (Uruguay) hundreds of megamammal bones -17 of 27 individuals were assigned to giant sloth (*Lestodon armatus)*- were recovered from the bed of the Vizcaino stream. On the basis of nine dates, Fariña et al. [31] proposed an age of the site between ~ 27,000 and 30,100 BP, with a pooled average between 32,298 and 31,219 cal ka BP”. These authors proposed that there two main evidence suggesting the human origin of the deposit: the mortality profile of the animals (most of them adults), and the identification of cut marks in the 5% of the identifiable bones [9]. The few lithic recovered at the site were considered “to have seemingly anthropogenic features” [9]. After the analysis and interpretations of the site were published, the site has been subject of criticism by several authors [32, 33]. In a previous publication [34], two of us pointed out the main problems of the site, which are summarized in the following lines: a). The site is located where the Vizcaino stream becomes deeper and form a natural pond on a silicified sandstone (Figure 1a in [31]). Such conditions (a depression with hydraulic energy) would have been a natural trapping device for carcasses, part of carcasses or disarticulated bones [35]. The presence of coarse grain related to a high energy event (see supplementary material in [31]) make questionable the idea that fluvial agency has to be ruled as the main responsible for the formation of the deposit [31]. b) Neither the human origin nor the spatial association of lithics are clear enough to be considered highly probable. c) The mortality profile of the animals is not necessarily an unequivocal proof of the human agency in the site. There are several studies showing than several natural agents can create a deposit with predominance of adult individuals from a single species [35]. d) The interpretation of marks on some bones as “the result of the action of human tools” [31] has not been sufficiently supported in spite of the serious efforts of the authors. The marks observed in the bone assemblage could have been produced by different taphonomic agents and process which generate similar bone modifications (equiffinality) [36, 37, 38, 39]. On the basis of the points referred above, and at its present stage we still see Arroyo del Vizcaino more as “an example of natural processes mimicking human presence”, one of the alternative proposed by the authors [31] than as a correlate of human agency.

*Lapa Vermelha and Toca do Gordo do Garrincho*

Lapa Vermelha and Toca do Gordo do Garrincho sites gave human remains with proposed ages of ca. 13,200-13,500 and 15,000-16,000 cal years BP. The famous dates of the Hominid 1 from Lapa Vermelha IV -also known as Luzia- in north-eastern Brazil come from charcoal from overlying and underlying levels as dated by Laming-Emperaire [10]. Since the skeleton seems to have been intentionally buried, (and therefore not penecontemporaneous with dated sediments) these dates might not bracket the age of the human remains. No direct collagen dates have been obtained from this skeleton and a reported age of ca. 10,300-10,500 BP was obtained from carbon found in the acid washes of the sample treatment [10]. In the case of Toca do Gordo do Garrincho [11], one sample yielded an age of 12,210 ± 40 14C yrs BP (ca. 14,000 cal yr BP,) but it was obtained from carbon recovered from acid washes of a combined sample of two human teeth recovered from different excavation levels.

Finally, dates form some well-known South American sites such as El Abra and Taima Taima etc, excavated long time ago have not been included basically for four reasons a) in same cases the association between the dated sample and the cultural material is not clear; certainly geoarchaeological and taphonomic studies are needed. b) same dates have been obtained from dispersed charcoal c) none of these dates were re-dated with modern and more accurate techniques (e.g. AMS, pre-treated with XAD or ultrafiltration, etc d) In some cases, based of detailed analysis, the cultural status of the lithics have been consistently questioned [40]

**References**

1. Parenti FM, Fontugue M, Guerin C. Pedra Furada in Brazil and its ‘presumed’ evidence: limitations and potential of the available data. Antiquity. 1996;70:416–21.
2. Lahaye C, Guérin G, Boëda E, Fortugne M, Hatté E, et al. New insights into a late-Pleistocene human occupation in America: the Vale da Pedra Furada complete chronological study. Quat. Geochronol. 2015;30:445-451.
3. Boëda E, Rocca R, Da Costa A, Fontugne M, Hatté C, et al. New Data on a Pleistocene Archaeological Sequence in South America: Toca do Sítio do Meio, Piauí, Brazil. PaleoAmerica. 2016; 2:286-302.
4. Lahaye C, Hernández M, Boëda E, Felice GD, Guidon N, et al. Human occupation in South America by 20,000 BC: the Toca da Tira Peia site, Piauí, Brazil. J. Archaeol. Sci. 2013;40:2840-2847.
5. Vialou AV, Ed., Pré-história do Mato Grosso, São Paulo: Edusp; 2005.
6. Pino M, Chávez-Hoffmeister M, Navarro-Harris X, Labarca R, The late Pleistocene Pilauco site, Osorno, south-central Chile. Quat. Int.2013;299:3-12.
7. Dillehay TD, Ocampo C, Saavedra J, Oliveira Sawakuchi A, Vega RM, Pino M, et al. New archaeological evidence for an early human presence at Monte Verde, Chile. PLoS ONE. 2015;10(11):e0141923. doi:10.1371/journal.pone.0141923.
8. Dillehay TD, Ocampo C, Saavedra J, Pino M, Scott-Cummings L, Kovácik P. et al*.*, New excavations at the late Pleistocene site of Chinchihuapi I, Chile. Quat. Res. 2019;92:70-80.
9. Fariña RA. Bone surface modifications, reasonable certainty, and human antiquity in the Americas: The case of the arroyo del Vizcaíno site. Am. Ant. 2015;80(1):193-200.
10. Neves WA, Powell JF, Ozolins EG. Modern human origins as seen from the peripheries. J. Hum. Evol. 1999;37:129-133.
11. Guidon N, Peyre E, Guerin C, Coppens Y, Resultados da datação de dentes humanos da Toca do Garrincho, Piauí—Brasil. Clio. 2000;14:75–86.
12. Guidon N, Delibrias G. Carbon-14 point to man in the Americans 32,000 years ago. Nature. 1986; 321 (6072):769–771.
13. Parenti F. Le gisement quaternaire de la Toca do Boqueirão da Pedra Furada (Piaui, Brésil): stratigraphie, chronologie, évolution culturelle. Paris: Editions Recherches sur Les Civilisations; 2001.
14. Parenti F. Pedra Furada, Archaeology of. In: Encyclopedia of Global Archaeology. London: Springer Science+Business Media; 2014, p. 5842-5855.
15. Meltzer D, Adovasio J, Dillehay TD. On a Pleistocene human occupation at Pedra Furada, Brazil. Antiquity 1994;68:695–714.
16. Proffitt T, Luncz LV, Falótico T, Ottoni EB, de la Torre I, et al. Wild monkeys flake stone tools. Nature 2016;539: 85–88.
17. Luncz LV, Falótico T, Pascual-Garrido A, Corat C, Mosley H, et al. Wild capuchin monkeys adjust stone tools according to changing nut properties. Sci. Reports. 2016;6:33089.
18. Boëda E, Lourdeau A, Lahaye C, Felice GD, Viana S, et al. In: Graf K, Ketron CV, Waters MR, editors. Paleoamerican Odyssey. Texas: A&M Texas University press; 2013. p. 425-445.
19. Fiedel SJ. Did Monkeys Make the Pre-Clovis Pebble Tools of Northeastern Brazil? PaleoAmerica. 2017;3:6-12.
20. Boëda E, Clemente-Conte I, Fontugne M, Lahaye C, Pino M, Daltrini Felice G, et al. A new late Pleistocene archaeological sequence in South America: the Vale da Pedra Furada (Piaui, Brazil). Antiquity. 2014;88:927–941.
21. Vialou D, Benabdelhadi M, Feathers J, Fontugne M, Vialou AV. Peopling South America's centre: the late Pleistocene site of Santa Elina. Antiquity 91. 2017; 358:865-884.
22. A. Vialou V. 2005 *Pré-História do Mato Grosso. Volumen 1: Santa Elina*. Editorial USP, Sao Paulo.
23. Dillehay TD. Monte Verde: A Late Pleistocene Settlement in Chile. Volume II: The Archaeological Context. Washington, D.C.: Smithsonian Institution Press; 1997.
24. Labarca R, Recabarren OP, Canales-Brellenthin P, Pino M. The gomphotheres (proboscidea: Gomphotheriidae) from Pilauco site: Scavenging evidence in the Late Pleistocene of the Chilean Patagonia. Quat. Int. 2014;352:75-84.
25. Labarca RL, Pino M, Recabarren O. Los Lamini (Cetartiodactyla: Camelidae) extintos del yacimiento de Pilauco (Norpatagonia chilena): aspectos taxonómicos y tafonómicos preliminares. Estudios Geológicos. 2013;69(2):255-269.
26. González E, Prevosti FJ, Pino M. Primer registro de Mephitidae (Carnivora: Mammalia) para el Pleistoceno de Chile. Magallania. 2010;38(2):239–248.
27. Recabarren O, Pino M, Alberdi M. La Familia Gomphotheriidae en América del Sur: Evidencia de molares al norte de la Patagonia chilena. Estudios Geológicos. 2014;70:1–12.
28. Pino M, Abarzúa AM, Astorga G, Martel-Cea A, Cossio-Montecinos N, et al. Sedimentary record from Patagonia, southern Chile supports cosmic-impact triggering of biomass burning, climate change, and megafaunal extinctions at 12.8 ka. Sci. Reports. 2019;9(1):4413.
29. Navarro‐Harris X, Pino M, Guzmán‐Marín P, Lira MP, Labarca R, et al. The procurement and use of knappable glassy volcanic raw material from the late Pleistocene Pilauco site, Chilean Northwestern Patagonia. Geoarchaeology. 2019: doi.org/10.1002/gea.21736.
30. Moreno K, Bostelmann JE, Macías C, Navarro-Harris X, De Pol-Holz R, et al. A late Pleistocene human footprint from the Pilauco archaeological site, northern Patagonia, Chile. PloS one. 2019;14:e0213572.
31. Fariña RA, Tambusso PS, Varela L, Czerwonogora A, Di Giacomo M, Musso M, Bracco-Boksar R and Gascue A. Arroyo del Vizcaíno, Uruguay: A fossil-rich 30-ka old megafaunal locality with cut-marked bones. Proceedings of the Royal Society B 2014,281:20132211.
32. Borrero LA. Ambiguity and debates on the Early Peopling of South America. Paleoamerica 2016,2(1):11‒21.
33. Suárez R., Borrero L.A, Borrazzo K, Ubilla M, Martínez S, and Perea D. Archaeological evidences are still missing: a comment on Fariña et al., Arroyo del Vizcaíno site, Uruguay. Proceedings Royal Society B 2014;281: 20140449.
34. Politis G, and Prates, L. Clocking the arrival of Homo sapiens in the Southern Cone of South America. In: New Perspectives on the Peopling of the Americas, Words Bones Genes Tools: DFG Center for Advanced Studies Series, Vol 1, Harvati K, Jäger G, and Centeno H, editors. Tübingen: Kerns Verlag; 2018, p. 79-106.
35. Rogers RR, and Kidwell SM. A conceptual framework for the genesis and analysis of vertebrate skeletal concentrations. In: Bonebeds: Genesis, Analysis, and Paleobiological Significance, Rogers RR, Eberth DA, and Fiorillo AR, editors. University of Chicago press: Chicago; 2007, p. 1‒63.
36. Behrensmeyer, A. K., K. D. Gordon, and G. T. Yanagi. Trampling as a cause of bone surface damage and pseudo-cutmarks. Nature 1986;317(27):768-771.
37. Domínguez-Rodrigo M, De Juana S, Galán AB, and Rodríguez M. A new protocol to differentiate trampling marks from butchery cut marks. Journal of Archaeological Science 2009;36:2643‒654.
38. Fernández-Jalvo Y, and Andrews, P. Experimental effects of water abrasion on bone fragments. Journal of Taphonomy 2003;1(3):147‒63.
39. Olsen, SL, and Shipman, P. Surface modification on bone: trampling versus butchery. Journal of Archaeological Science 1988;15(5):535‒53
40. Muttillo, B., Lembo, G., Rufo, E., Peretto, C., and Lleras Pérez, R., 2017. Revisiting the oldest

known lithic assemblages of Colombia: a review of data from el Abra and tibitó

(cundiboyacense plateau, eastern Cordillera, Colombia). Journal of Archaeolical. Science.:

Report 2017 13: 455–465.
